# Evaluation of the anticarcinogenic potential of the endophyte, *Streptomyces* sp. LRE541 isolated from *Lilium davidii* var. *unicolor* (Hoog) Cotton

**DOI:** 10.1186/s12934-021-01706-z

**Published:** 2021-12-04

**Authors:** Aiai Ma, Kan Jiang, Bin Chen, Shasha Chen, Xinge Qi, Huining Lu, Junlin Liu, Xuan Zhou, Tan Gao, Jinhui Li, Changming Zhao

**Affiliations:** 1 State Key Laboratory of Grassland Agro-Ecosystems, School of Life Sciences, Lanzhou University, Lanzhou, 730000 Gansu China; 2 Yuzhong Mountain Ecosystem Field Observation and Research Station, Lanzhou University, Lanzhou, 730000 Gansu China; 3 Department of Animal and Biomedical Sciences, School of Life Sciences, Lanzhou University, Lanzhou, 730000 China; 4 Life Science and Engineering College of Northwest University for Nationalities, Lanzhou, 730000 China; 5 College of Agronomy, Gansu Agricultural University, Lanzhou, 730070 China

**Keywords:** Endophytic *Streptomyces* sp. LRE541, Bioactive metabolites, Indole diketopiperazine alkaloid, Anthraquinones, Anticancer activity, Cell apoptosis, Cell cycle

## Abstract

**Background:**

Endophytic actinomycetes, as emerging sources of bioactive metabolites, have been paid great attention over the years. Recent reports demonstrated that endophytic streptomycetes could yield compounds with potent anticancer properties that may be developed as chemotherapeutic drugs.

**Results:**

Here, a total of 15 actinomycete-like isolates were obtained from the root tissues of *Lilium davidii* var. *unicolor* (Hoog) Cotton based on their morphological appearance, mycelia coloration and diffusible pigments. The preliminary screening of antagonistic capabilities of the 15 isolates showed that isolate LRE541 displayed antimicrobial activities against all of the seven tested pathogenic microorganisms. Further in vitro cytotoxicity test of the LRE541 extract revealed that this isolate possesses potent anticancer activities with IC_50_ values of 0.021, 0.2904, 1.484, 4.861, 6.986, 8.106, 10.87, 12.98, and 16.94 μg/mL against cancer cell lines RKO, 7901, HepG2, CAL-27, MCF-7, K562, Hela, SW1990, and A549, respectively. LRE541 was characterized and identified as belonging to the genus *Streptomyces* based on the 16S rRNA gene sequence analysis. It produced extensively branched red substrate and vivid pink aerial hyphae that changed into amaranth, with elliptic spores sessile to the aerial mycelia. To further explore the mechanism underlying the decrease of cancer cell viability following the LRE541 extract treatment, cell apoptosis and cell cycle arrest assays were conducted in two cancer cell lines, RKO and 7901. The result demonstrated that LRE541 extract inhibited cell proliferation of RKO and 7901 by causing cell cycle arrest both at the S phase and inducing apoptosis in a dose-dependent manner. The chemical profile of LRE541 extract performed by the UHPLC-MS/MS analysis revealed the presence of thirty-nine antitumor compounds in the extract. Further chemical investigation of the LRE541 extract led to the discovery of one prenylated indole diketopiperazine (DKP) alkaloid, elucidated as neoechinulin A, a known antitumor agent firstly detected in *Streptomyces*; two anthraquinones 4-deoxy-*ε*-pyrromycinone (1) and epsilon-pyrromycinone (2) both displaying anticancer activities against RKO, SW1990, A549, and HepG2 with IC_50_ values of 14.96 ± 2.6 − 20.42 ± 4.24 μg/mL for (1); 12.9 ± 2.13, 19.3 ± 4.32, 16.8 ± 0.75, and 18.6 ± 3.03 μg/mL for (2), respectively.

**Conclusion:**

Our work evaluated the anticarcinogenic potential of the endophyte, *Streptomyces* sp. LRE541 and obtained one prenylated indole diketopiperazine alkaloid and two anthraquinones. Neoechinulin A, as a known antitumor agent, was identified for the first time in *Streptomyces*. Though previously found in *Streptomyces*, epsilon-pyrromycinone and 4-deoxy-*ε*-pyrromycinone were firstly shown to possess anticancer activities.

**Graphical Abstract:**

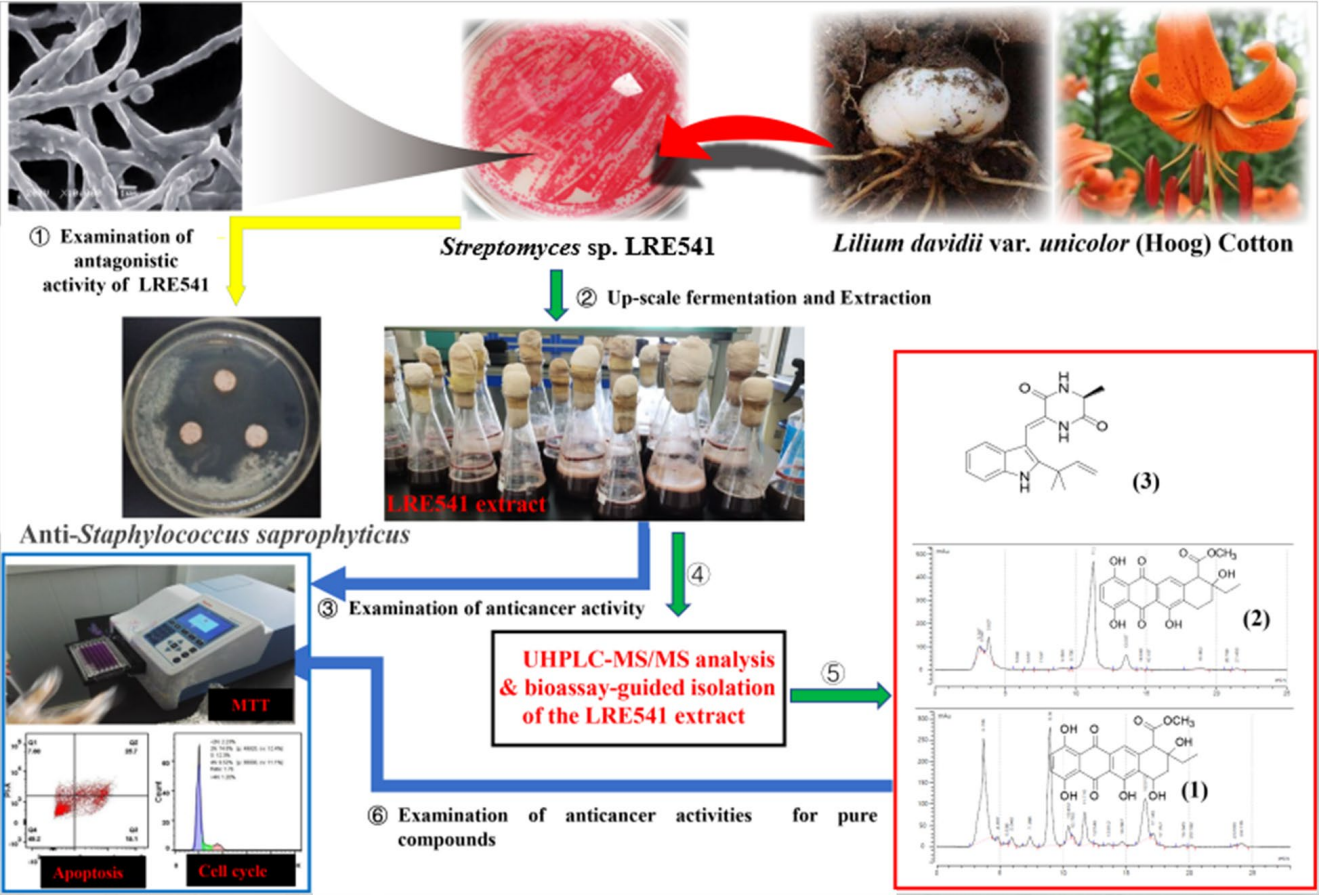

**Supplementary Information:**

The online version contains supplementary material available at 10.1186/s12934-021-01706-z.

## Introduction

Although major progress has been achieved in cancer therapy for the past few decades, cancer remains a serious public health threat [1]. Chemotherapy is one of the common therapeutic approaches for controlling cancers. Unfortunately, most patients eventually relapse and develop drug resistance [2, 3]. On this account, a continuous supply of novel drugs with high effectiveness and safety is urgently needed. Besides, drug-induced apoptosis of malignant cells is a promising antitumor strategy with emerging evidence supporting its efficacy against various cancer types [4–6]. Thus, extensive and intensive studies on the underlying antitumor mechanisms of the drugs are also required.

The genus *Streptomyces*, with its vast distribution and innate capability of producing diverse bioactive secondary metabolites, has served as an important source of novel antibiotic candidates for decades [7–9]. Nowadays, exploiting streptomycetes from untapped or unique ecosystems may be an effective way to meet the everlasting demand for novel drugs and other biomolecules, which have been preferred attributing to their potent therapeutic applications and desired pharmacokinetic properties for clinical uses and served as precursors of drug semi-synthesis or the template of drug chemical synthesis [10, 11]. Over the past decade, endophytic streptomycetes from medicinal plants in various ecotopes, as relatively unexploited fascinating sources of novel natural products, have been explored extensively and gained some remarkable results. For example, reports covering new endophytic *Streptomyces* species and their novel secondary metabolites along with antimicrobial and antioxidant activities have sprung up [11–14]. Moreover, endophytic streptomycetes have been reported to possess anticancer activities as well. Although such reports are sporadic compared to marine actinomycetes, the anticancer effects or cytotoxic activities of endophytic streptomycetes are comparable to those of their marine counterparts, even stronger [15, 16]. In addition, it is widely accepted that medicinal plants are rich sources of precious bioactive compounds, and increasing evidence indicates that endophytic actinomycetes may participate in the metabolic pathways of their host plants and obtain some genetic information to yield bioactive compounds similar to their host plants [17, 18]. These findings suggest that bioprospecting of endophytic streptomycetes from medicinal plants may be a good choice for anticarcinogens discovery.

*Lilium davidii* var. *unicolor* (Hoog) Cotton (commonly called Lanzhou lily), a famous healthcare edible medicinal plant rich in amino acid, vitamins, glycosides, alkaloids, and polysaccharides, possesses antioxidant activities [19]. Accordingly, the actinomycetes from the plants may develop adaptive strategies and yield chemically unique secondary metabolites. However, there is no report concerning the anticancer activities of *Streptomyces* species from *L*. *davidii* var. *unicolor* (Hoog) Cotton in vitro. Given the immense potential of the secondary metabolites of endophytic actinomycetes for pharmaceutical applications, we isolated and characterized actinomycetes from the root tissues of *L. davidii* var. *unicolor* (Hoog) Cotton, and investigated the antimicrobial capabilities of the isolates. The most potent isolate was screened for evaluating antitumor activities, as well as the inductive effects on apoptosis and cell cycle arrests of tumor cells. Further, the active compounds from the isolate were purified and characterized.

## Results

### Isolation of endophytic actinomycetes and screening for antimicrobial activities

On the basis of colonial morphology, mycelia coloration and diffusible pigments, fifteen actinomycete-like isolates with representative phenotypes were obtained from the root tissues of *L. davidii* var. *unicolor* (Hoog) Cotton**.** The preliminary screening of antimicrobial capabilities showed that two isolates had exhibited antagonistic activities against at least four of the tested pathogenic microorganisms (Additional file 1: Table S1), especially LRE541 isolate against all the tested Gram-positive/-negative bacteria and the yeast-like fungus with the maximum activity against *Staphylococcus saprophyticus* (inhibition zone of 21.33 mm diameter) (Table 1). Thus, LRE541 isolate was selected for up-scale fermentation and extraction of secondary metabolites for further assessments.

**Table 1:** Antimicrobial activities of isolate LRE541 against various pathogenic microorganisms

Test microorganisms	Inhibition zone (mm diameter)
Gram-positive bacteria	
*Staphylococcus aureus* (MRSA) ATCC25923	16.67 ± 2.31
*Diplococcus pneumoniae* (clinical isolate)	16 ± 2.65
*Enterococcus faecalis* (clinical isolate)	12.33 ± 0.58
*Staphylococcus saprophyticus* (clinical isolate)	21.33 ± 1.53
Gram-negative bacteria	
*Escherichia coli* ATCC25922	12.33 ± 1.53
*Pseudomonas aeruginosa* ATCC27853	12 ± 1.00
The yeast-like fungus	
*Candida albicans* ATCC66415	7.67 ± 0.78

### Phenotypic characteristics of isolate LRE541

**Fig. 1:**
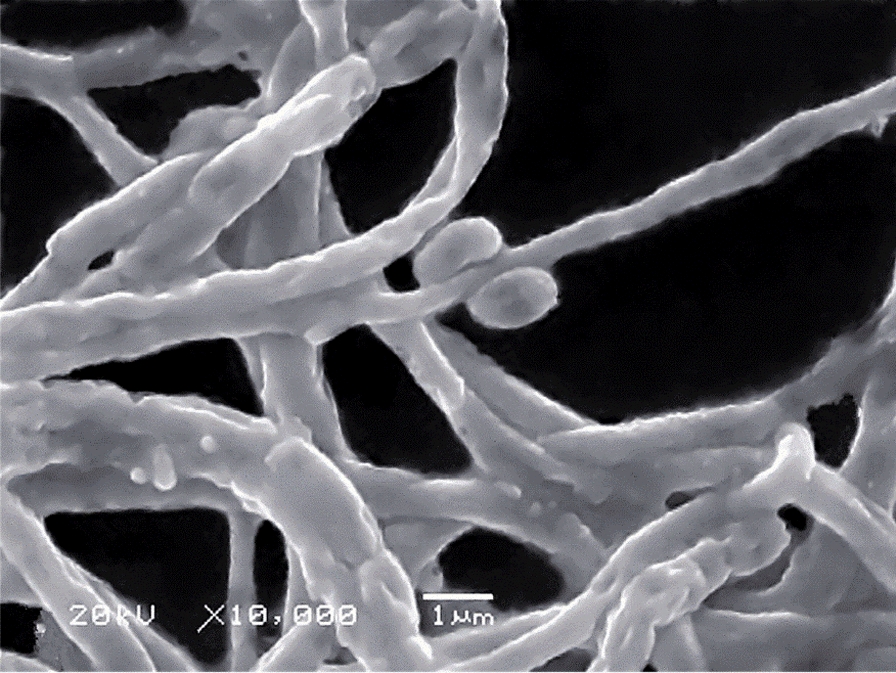
The scanning electron micrograph of *Streptomyces* sp. LRE541 cultured on the Gauze’s No. 1 medium for 2 weeks showing aerial mycelia and spores

The isolate LRE541 obtained from the root tissues of *L. davidii* var. *unicolor* (Hoog) Cotton was Gram-positive and aerobic. The cultural characteristics of LRE541 on various media were shown in Table 2. It grew well on all the tested media (ISP2–ISP7 and Gauze’s No. 1) with varying aerial and substrate mycelia colors on different media. Compared with ISPs, the diffusible pigment was only produced on the Gauze’s No. 1 medium. As presented under the scanning electron microscopy in Fig. 1, isolate LRE541 produced elliptic spores sessile to the aerial hyphae, which extensively branched and grew in segments with verrucous protrusions. As shown in Table 3, the growth of LRE541 was observed at the temperature range of 18–37 °C (optimum at 23 °C), and the pH range of 4–12; however, at NaCl concentration above 6% (w/v), no growth was observed. Isolate LRE541 was positive for cellulose utilization but negative for both methyl red test and H_2_S production. In the extracellular enzyme activity tests, the isolate LRE541 demonstrated to produce various enzymes such as urease, catalase, amylase, protease, and lipase. Furthermore, it was found that LRE541 had broad utilization of carbon and nitrogen sources (Table 3). These phenotypic properties of LRE541 were in close similarity with the genus *Streptomyces* as depicted by Barka et al. [20] in that they are prolific aerobic Gram-positive bacteria possessing extensively branched vegetative form and aerial hyphae and produce various water-soluble pigments.

**Table 2:** Cultural characteristics of *Streptomyces* sp. LRE541 on various media

Media	Growth	Color of colony mycelia		Diffusible pigment
		Aerial	Substrate	
ISP2	Good	Light pink to red	Orange-yellow to red	–
ISP3	Good	Bright red	Red	–
ISP4	Good	Pinky white	Pinky white	–
ISP5	Good	Red in white	Red in white	–
ISP6	Good	Transparent to pale violet red	Pale violet red	–
ISP7	Good	Brick red	Vivid red	–
Gause’s No. 1	Good	Vivid pink	Red	Claret-colored pigment

–, absent

**Table 3:** Physiological properties of *Streptomyces* sp. LRE541

Tests	Results	Tests	Results
Cellulose utilization	+	**Nitrogen sources utilization**	
MR test	−	Urea	+
H_2_S production	−	Glycine	+
**Extracellular enzyme activity**		Peptone	+
Urease	+	Maizena	−
Catalase	+	Tyrosine	+
Starch hydrolysis	+	Aspartic acid	−
Gelatin hydrolysis	+	Soybean meal	+
**Degradation of**		Ammonium sulfate	+
Tween 20	−	l-Proline	+
Tween 40	+	l-Arginine	+
Tween 80	+	l-α-Alanine	−
**Carbon sources utilization**		**Growth at pH**	
Xylose	++	pH 2	−
Starch	+	pH 4	+
Glucose	++	pH 6	+
Maltose	+++	pH 7	+++
Lactose	+++	pH 8	++
Sucrose	++	pH 10	++
Fructose	+	pH 12	+++
Mannose	++	**Growth at Temp**	
Trehalose	−	4–16 °C	−
Raffinose	−	18–20 °C	+
Arabinose	+	23 °C	+++
Rhamnose	+	28 °C	++
**Growth at NaCl (w/v)**		37 °C	+
0–6%	++	**Gram staining**	+

“−”, negative test; “+”, positive test/slight growth; “++”, well-growth; “+++”, very well growth

### 16S rRNA gene-based phylogenetic analysis

**Fig. 2:**
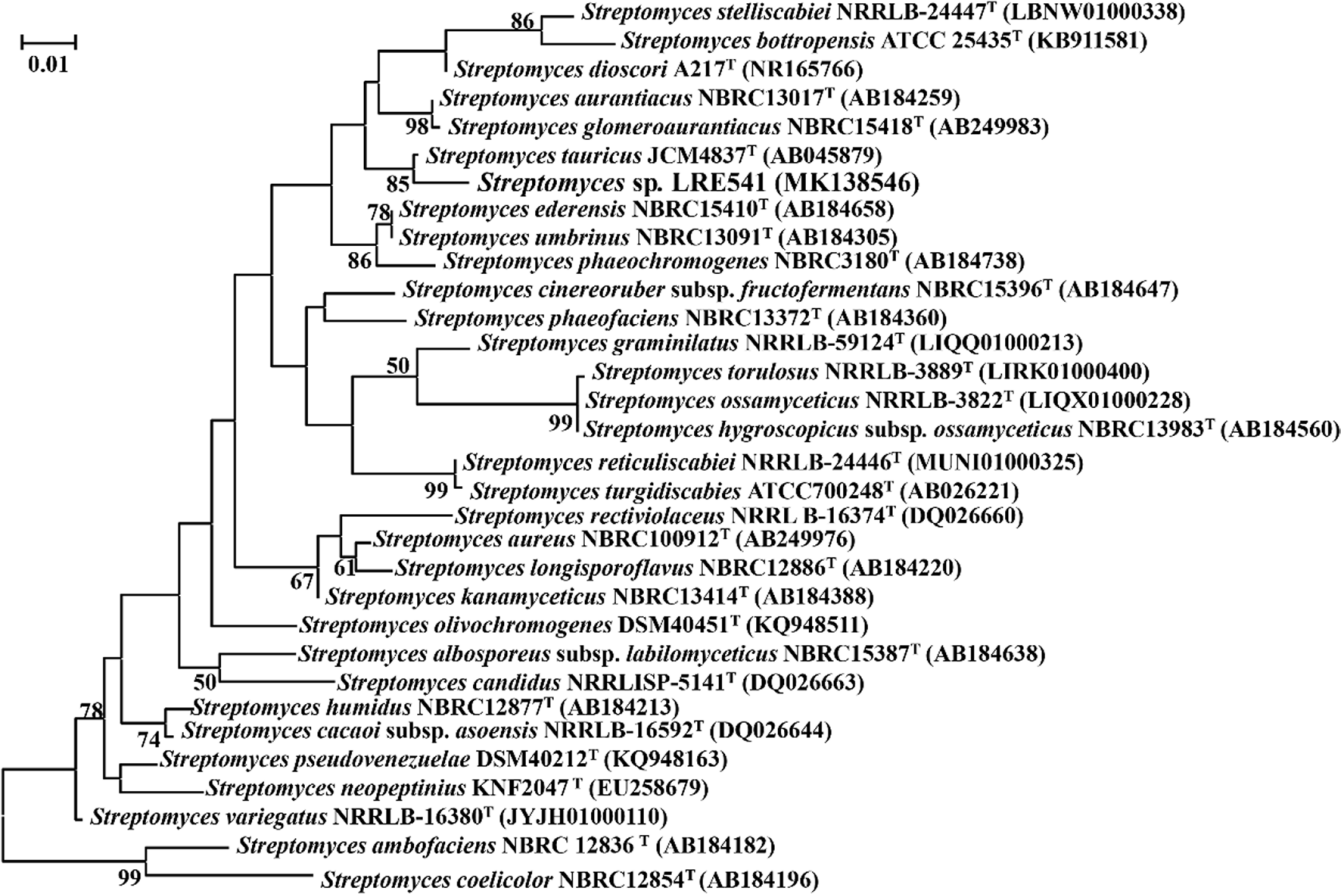
Maximum Likelihood tree exhibiting phylogenetic relationship between isolate LRE541 and the closely related representatives of *Streptomyces* spp. Only bootstrap values above 50% are present at the tree nodes. The scale bar denotes 0.01 substitutions per site

The almost complete 16S rRNA gene sequencing revealed that isolate LRE541 comprised 1471 bp, which was submitted in GenBank/EMBL/DDBJ under the accession number MK138546 (https://www.ncbi.nlm.nih.gov/nuccore/MK138546). The 16S rRNA gene sequence of LRE541 was aligned with those of the type strains retrieved from GenBank/EMBL/DDBJ databases. As presented in Fig. 2, the phylogenetic tree demonstrated that LRE541 formed a distinct phyletic line with the type strain *Streptomyces tauricus* JCM4837^T^ at bootstrap value of 85%, displaying the highest 16S rRNA gene sequence similarity value with *Streptomyces tauricus* JCM4837^T^ (98.81%), and followed by *Streptomyces ederensis* NBRC15410^T^ (98.45%), *Streptomyces dioscori* A217^T^ (98.25%), *Streptomyces aurantiacus* NBRC13017^T^ (98.18%), and *Streptomyces glomeroaurantiacus* NBRC15418^T^ (98.12%).

### Cytotoxicity of the LRE541 extract towards various cell lines

**Fig. 3:**
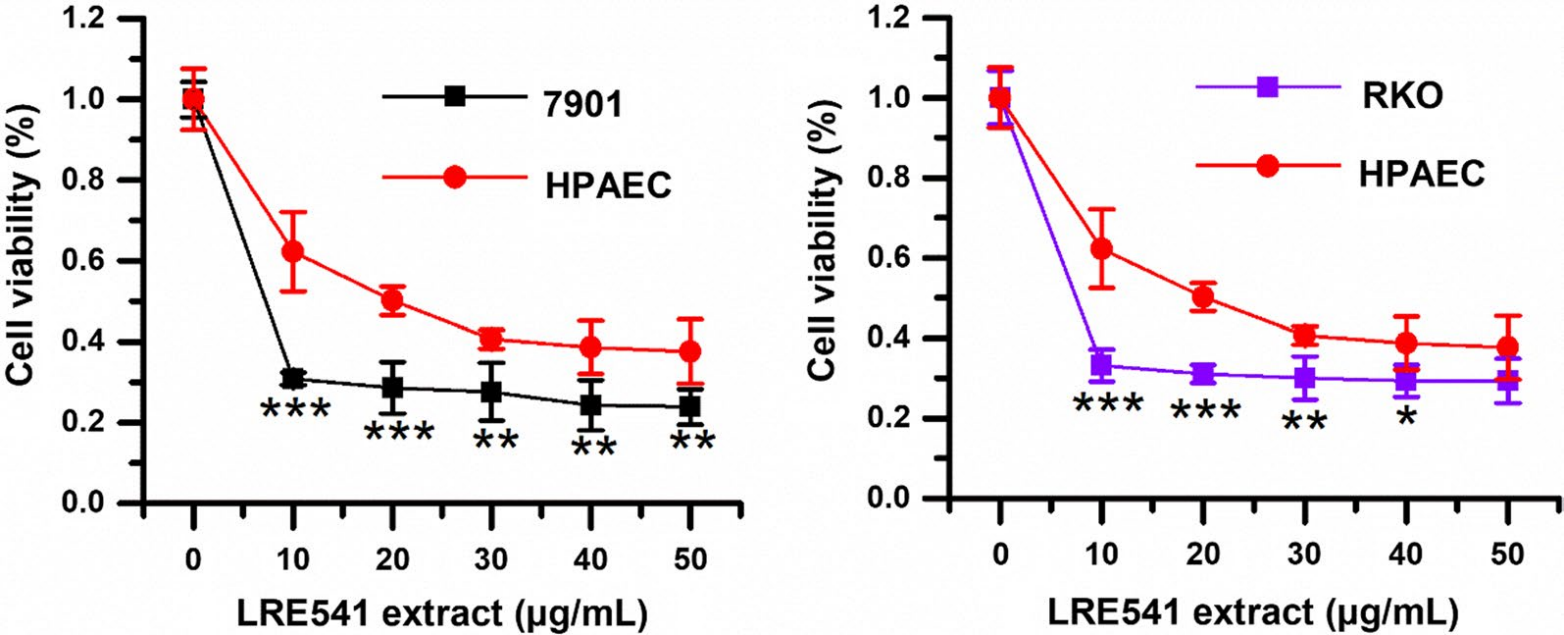
Sensitivity of various types of cell lines (7901, RKO and HPAEC) to the LRE541 extract. The three cell lines were incubated with increasing concentrations of the LRE541 extract for 48 h, and their viabilities were determined by the MTT method. *P < 0.05, **P < 0.01, ***P < 0.001 vs. the HPAEC cell line

We had determined the cytotoxicity of LRE541 extract towards a panel of cancer cell lines (A549, SW1990, HepG2, CAL-27, MCF-7, 7901, RKO, Hela, and K562) and one normal cell line human pulmonary artery endothelial cell (HPAEC). As illustrated in Table 4, the LRE541 extract exhibited cytotoxic activities against six cancer cell lines with IC_50_ values < 10 μg/mL, and against all of the cancer cell lines with IC_50_ values (0.021–16.94 μg/mL) < 20 μg/mL. Among the nine tested cancer cell lines, the LRE541 extract demonstrated the most potent efficacy towards RKO, followed by 7901 and HepG2 with IC_50_ values of 0.021, 0.29, and 1.484 μg/mL, respectively, after 48 h treatment. Compared to the cytotoxicity against HPAEC with IC_50_ value of 20.14 μg/mL, the LRE541 extract displayed a greater cytotoxicity towards RKO, 7901, and HepG2 in vitro. In conclusion, the LRE541 extract potently inhibited various cell types with a high preference for RKO and 7901. Thus, RKO and 7901 were opted to furtherly investigate the effect of LRE541 extract on cancer cells. As illustrated in Fig. 3, compared to HPAEC, the cell viabilities of RKO and 7901 dramatically decreased when the concentration of the LRE541 extract was within 10 μg/mL, and below 30% when reached 10 μg/mL.

**Table 4:** IC_50_ values of the LRE541 extract against various cell lines (μg/mL)

Cell types	IC_50_
Human colon cell RKO	0.02127
Human gastric adenocarcinoma 7901	0.2904
Human liver carcinoma cell HepG2	1.484
Human tongue cancer cell CAL-27	4.861
Human breast carcinoma cell MCF-7	6.986
Human chronic promyelocytic leukemia cell K562	8.106
Human cervical cancer cell Hela	10.87
Human pancreatic cancer cell SW1190	12.98
Human non-small cell lung cancer A549	16.94
Human pulmonary artery endothelial cell HPAEC (human normal cell)	20.14

### Induction of apoptosis in 7901 and RKO cell lines

**Fig. 4:**
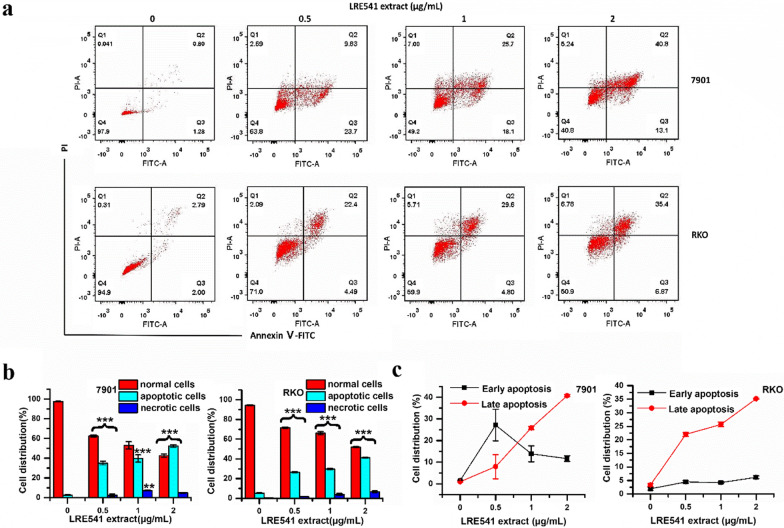
The LRE541 extract induces apoptosis in RKO and 7901 cell lines. **a** The apoptotic cells change in RKO and 7901 cell lines when treated with increasing concentrations of the LRE541 extract for 48 h by Annexin V and PI double-staining assay. **b** The quantification of necrotic cells, apoptotic cells and normal cells. The data are presented as the mean ± SD of three independent experiments. *P < 0.05, **P < 0.01, ***P < 0.001 vs. the control groups. **c** Early and late apoptotic cells of 7901 and RKO treated with varying concentrations of the LRE541 extract for 48 h

Inducing apoptosis and necrosis of tumor cells is the primary mechanism of chemotherapeutic drugs inhibiting tumors, and it is also one of the leading indicators for evaluating the efficacy of chemotherapeutic drugs [21, 22]. Herein, we quantitatively detected the cell death type triggered by the LRE541 extract in 7901 and RKO cell lines using the annexin V-FITC and PI double staining, which were presented in Fig. 4a. After the cells were processed with the LRE541 extract (2 μg/mL) for 48 h, FITC-positive cells accounted for ~ 50% and ~ 40% of the total cells in 7901 and RKO, respectively, suggesting that apoptosis was a major mechanism of the cytotoxicity of the LRE541 extract whether in 7901 or RKO cell line, and the LRE541 extract induced apoptotic cell death in a dose-dependent manner in both two cell lines (Fig. 4b). However, the apoptosis patterns of the RKO and 7901 were distinctly diverse. As demonstrated in Fig. 4c, for the 7901, the proportion of early apoptotic cells was higher than that of late apoptotic cells at low concentration of the LRE541 extract; however, the number of early apoptotic cells gradually decreased as the concentration increased, while the number of late apoptotic cells sharply increased with the increased concentration of LRE541 extract. In contrast, for the RKO cell line, the late apoptotic cells were dominant at first and displayed a dose-dependent manner, while the number of early apoptotic cells slightly increased with the increasing concentration.

### The LRE541 extract inhibits the cell cycles of 7901 and RKO cell lines

**Fig. 5:**
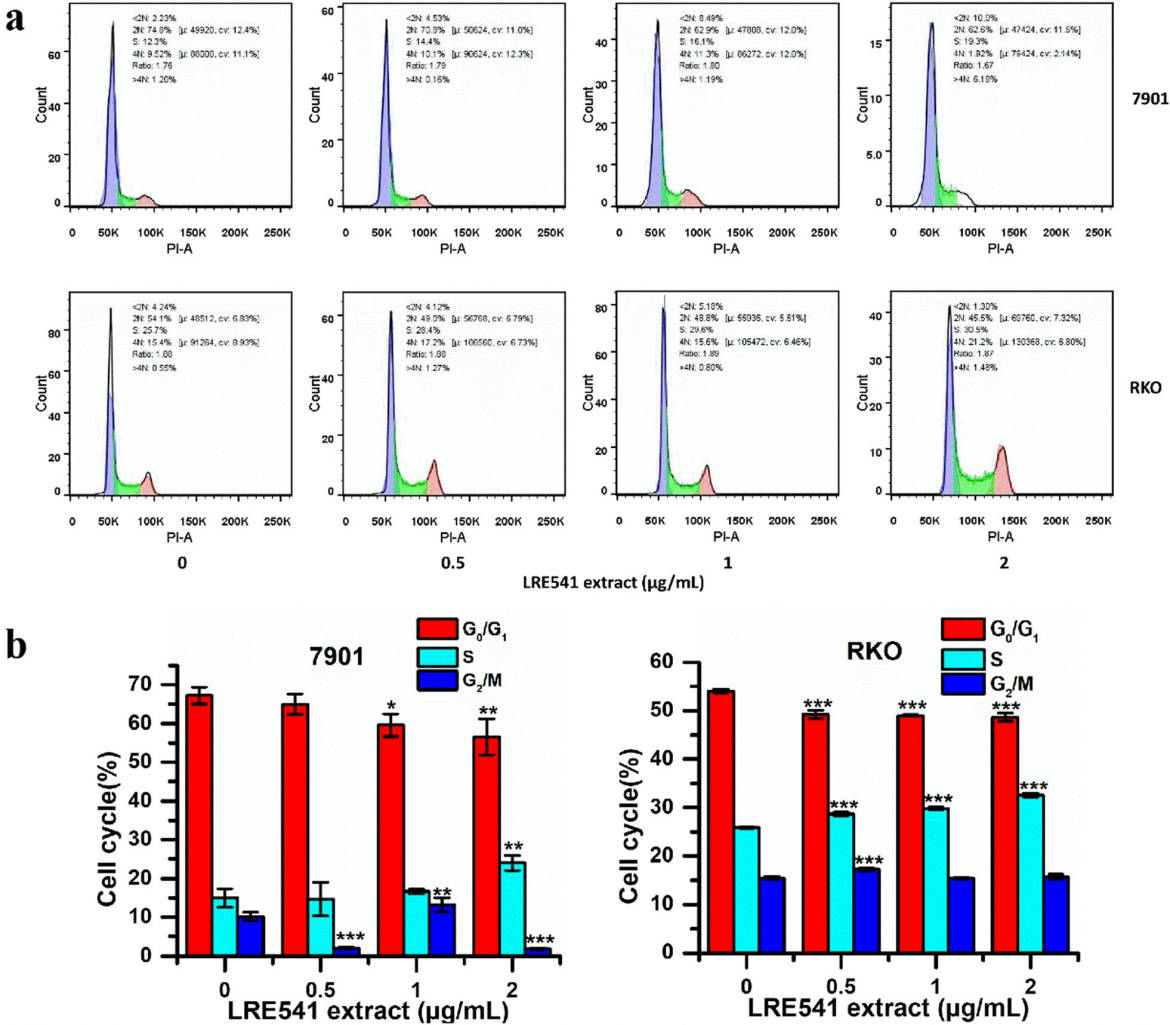
Effects of the LRE541 extract on cell cycles of RKO and 7901. **a** Cell cycle progressions of 7901 and RKO when treated with increasing concentrations of the LRE541 extract for 48 h. **b** Quantification of living cells distributing in three distinct phases of the cell cycle (G_0_/G_1_, S, and G_2_/M phase). The data are shown as the mean ± SD of three independent experiments. *P < 0.05, **P < 0.01, ***P < 0.001 vs. the control groups

Flow cytometric analysis of DNA showed a dose-dependent accumulation of cells in the S phase of the cell cycle both in 7901 and RKO cell lines, with a concomitant decrease in the proportion of cells in the G_0_/G_1_ phase when treated with a concentration gradient of the LRE541 extract for 48 h, indicating the LRE541 extract blocked the cell cycle of 7901 and RKO in S phase (Fig. 5a, b).

### Chemical profiling of the LRE541 extract using UHPLC-MS/MS analysis

To examine the compounds that may be responsible for its antineoplastic properties, the LRE541 extract was subjected to ultra-high performance liquid chromatography-tandem mass spectrometry (UHPLC-MS/MS) analysis (Additional file 1: Fig. S1), which detected the presence of approximately 700 compounds in the LRE541 extract. More than a seventh of the compounds were documented to exhibit various biological activities, including thirty-nine antitumor compounds, ten antioxidant compounds, and sixteen antimicrobial compounds. The detailed information of the sixty-five compounds, including retention time, molecular formula, molecular weight, and relative ratio, was listed in Additional file 1: Table S2 and their chemical structures were presented in Additional file 1: Fig. S2.

### Structure elucidation and cytotoxicity of compounds from LRE541 extract

**Fig. 6:**
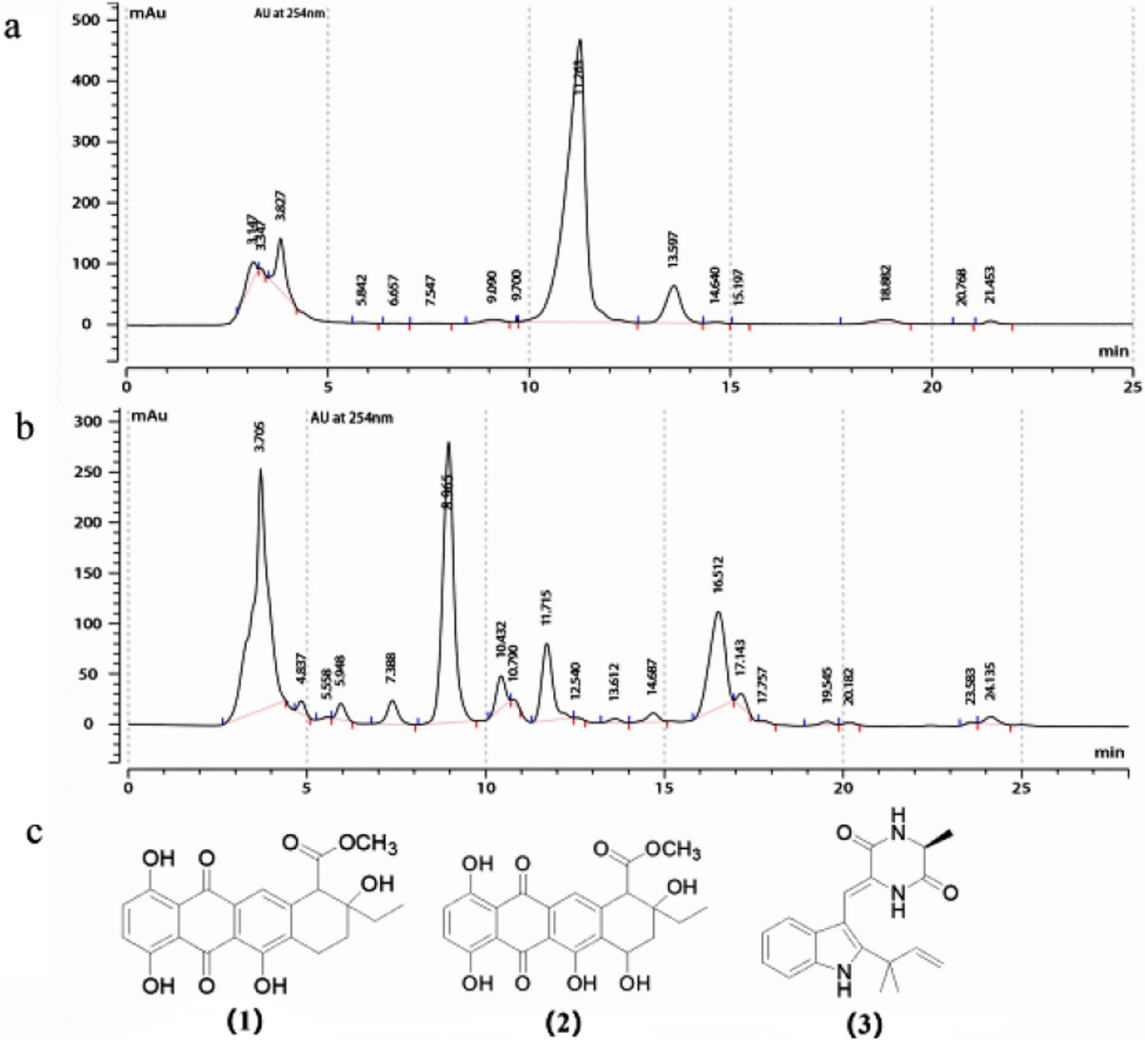
**a**, **b** Isolation and purification of 4-deoxy-ε-pyrromycinone at 11.263 min and epsilon-pyrromycinone at 8.965 min, respectively, by semi-HPLC; **c** chemical structures of the three pure compounds

The active fraction C4 (anticancer activity against SW1990 with IC_50_ value of 31.4 μg/mL) was subjected to silica gel column, sephadex LH-20 column, and compound 3 was obtained from preparing thin-layer chromatography (TLC). The fraction C3 (anticancer activity against SW1990 with IC_50_ value of 81.35 μg/mL) was re-chromatographed by semi-preparative HPLC till showing pure compounds at 11.263 min and 8.965 min to obtain compound 1 and 2 as seen in Fig. 6a, b. Based on 1D and 2D NMR spectroscopic analyses (Table 5, Additional file 1: Figs. S3–S10) and by comparison with those reported in the literatures, the three pure components were identified as two anthraquinone compounds 4-deoxy-*ε*-pyrromycinone (1), epsilon-pyrromycinone (2), and neoechinulin A (3), a prenylated diketopiperazine alkaloid. The chemical structures of the three compounds were depicted in Fig. 6c.

**Table 5:** ^13^C NMR spectroscopic data of compounds (1)–(3) [400 MHz,*δ* (ppm)] purified from the LRE541extract

(1) (CDCl_3_)		(2) (CDCl_3_)		(3) ((CD_3_)_2_CO)	
Position	*δ*_C_	Position	*δ*_C_	Position	*δ*_C_
1	159	1	158.6	2	144.8
2	130	2	130.3	3	104.4
3	129.7	3	129.8	3a	126.6
4	157.9	4	158	4	119.7
5	191.2	5	191.1	5	120.7
6	161.8	6	161.2	6	127.3
7	20.3	7	62.6	7	110.7
8	28.8	8	32	7a	136.2
9	71.8	9	70.1	8	112.4
10	57.3	10	57.6	9	122.2
11	121	11	120.8	10	160.3
12	186.5	12	185.3	12	52.2
13	171.7	13	170.3	13	166.9
14	53.4	14	53	15	40.1
15	32.6	15	34.7	15a × 2	27.9
16	7	16	6.3	16	146
4a	113	4a	112.5	17	112.3
5a	131.3	5a	132.2		
6a	134.5	6a	132.8		
10a	142.1	10a	142.6		
11a	114	11a	114.9		
12a	112.9	12a	112.3		

Neoechinulin A had been repeatedly reported to be cytotoxic to at least seven cancer cell lines [23, 24]. Thus, we mainly evaluated the antitumor activities of compounds (1) and (2), which showed cytotoxic activities against RKO, A549, HepG2, and SW1990 with IC_50_ values in the range of 14.96 ± 2.6 to 20.42 ± 4.24 μg/mL for (1) and 12.9 ± 2.13, 19.3 ± 4.32, 16.8 ± 0.75, and 18.6 ± 3.03 μg/mL for (2) (Table 6). The antitumor activities of 4-deoxy-*ε*-pyrromycinone and epsilon-pyrromycinone both displayed a preference for RKO cell line, slightly superior to cisplatin.

**Table 6:** IC_50_ values of the compounds 4-deoxy-*ε*-pyrromycinone (1), epsilon-pyrromycinone (2), and cisplatin (DDP) against various cancer cell lines (μg/mL). The data are shown as the mean ± SD of three independent experiments

Compounds	A549	HepG2	SW1990	RKO
(1)	19.55 ± 5.2	20.42 ± 4.24	17.87 ± 2.73	14.96 ± 2.6
(2)	16.8 ± 0.75	18.6 ± 3.03	19.3 ± 4.32	12.9 ± 2.13
DDP	12.8 ± 0.37	13.3 ± 1.2	17.1 ± 2.8	16.72 ± 3.5

## Discussion

Our study got fifteen actinomycete-like isolates from the root tissues of *L. davidii* var. *unicolor* (Hoog) Cotton. The preliminary screening of antagonistic capabilities of the 15 isolates found that isolate LRE541 showed antimicrobial activities against all of the tested pathogenic microorganisms. Thus, LRE541 isolate was furtherly evaluated for the anticarcinogenic potential against a variety of malignancies.

### Description of *Streptomyces* sp. LRE541

The isolate LRE541, well-characterized by the comparative analysis of 16S rRNA gene sequence, was assigned to *Streptomyces* sp. The phylogenetic relationship demonstrated that isolate LRE541 formed a distinct branch with the highest 16S rRNA gene sequence similarity of 98.81% to the type strain *Streptomyces tauricus* JCM4837^T^. Phenotypically, isolate LRE541 grew well on all the tested media with diverse aerial and substrate mycelia but produced red diffusible pigment only on the Gauze’s No. 1 medium. The extracellular enzyme tests found that LRE541 had the potential to yield various enzymes such as protease, amylase, and lipase, which are industrially important. In addition, LRE541 could tolerate a high pH value up to 12.0, a salinity of 6% (w/v) NaCl, and a temperature up to 37 °C. In sum, these physiological traits are similar to the characteristics of streptomycetes that they are prolific and possess the high adaptive capability for surviving in many unique niches [25–27], what’s more, reflecting the physiological flexibility of *Streptomyces* isolate to adverse environmental conditions [28, 29]. Furthermore, the availability of a broad spectrum of carbon and nitrogen sources plays a vital role in producing diverse secondary metabolites by *Streptomyces* sp. [30]. Here, isolate LRE541 also exhibited the capability to utilize a wide range of carbon and nitrogen sources. This data provided an overview of the metabolite profile of LRE541, potentially serving as references for future research concerning fermentation optimization for a higher yield of the desirable bioactive metabolites.

### Cytotoxic activities of the extract and pure compounds from LRE541

In view of the remarkable antimicrobial activities and physiological capabilities mentioned above, the secondary metabolites (LRE541 extract) of LRE541 were examined against nine representative human malignant tumors in vitro. The result revealed that the LRE541 extract showed cytotoxic activity towards all of the tumor cell lines with IC_50_ < 20 μg/mL, which is within the cut-off point of cytotoxicity criteria recommended by the National Cancer Institute (NCI) for screening the cytotoxicity of crude plant extracts [31, 32]. Moreover, the LRE541 extract exhibited cytotoxic activity against approximately 70% of the examined cancer cell lines with IC_50_ < 10 μg/mL, and great antitumor potential against RKO, 7901 with IC_50_ values of only 0.021 and 0.29 μg/mL, respectively. The potent antineoplastic capacities of the LRE541 extract suggested that the presence of bioactive agents in the extract may account for it. Based on the UHPLC-MS/MS analysis, the LRE541 extract was detected to contain thirty-nine antitumor-, ten antioxidant- and sixteen antimicrobial-compounds documented in numerous studies. Taking the metabolite profile as a reference and combining it with bioassay-guided isolation of the LRE541 extract, we obtained three bioactive compounds. Neoechinulin A, a prenylated indole diketopiperazine (DKP) alkaloid, was derived from the fungus *Aspergillus* species [33], and subsequently, it was isolated from a higher plant *Bridelia ferruginea* [23] and a marine-derived fungus *Microsporum* sp. [34]. Neoechinulin A had been demonstrated to be a valuable cancer cell growth inhibitor against seven cancer cell lines (KM20L2, NCI-H460, SF-295, BXPC-3, DU-145, OVCAR-3, and P388) with GI_50_ values in the range of 0.19–0.27 μg/mL [23]. Moreover, neoechinulin A had been shown to inhibit Hela cell proliferation by inducing cell apoptosis through down-regulating of Bcl-2 expression, up-regulating of Bax expression, and activating the caspase-3 pathway [24]. In addition, neoechinulin A was characterized by several bioactivities, including anti-oxidant [33], anti-inflammatory and anti-fouling activities [35, 36]. So far, prenylated indole diketopiperazines as important biological agents or their precursors were often detected in fungi, while neoechinulin A had been found in fungi and plants only, our study adds *Streptomyces* sp. as a new source for prenylated indole diketopiperazine production. Recently, anthraquinones were frequently detected in streptomycetes isolated from extreme or special environments such as marine, termites and plant tissues [34, 37, 38], exhibiting diverse bioactivities including antineoplastic activities. For example, grincamycin C and D derived from marine *Streptomyces lusitanus* SCSIO LR32 displayed cytotoxic activities against HepG2 and SW-1990 with IC_50_ values of 31 μM, 9.7 μM for C, 31 μM and 22 μM for D, respectively [39]. Termstrin A from termite-associated *Streptomyces* sp. BYF63 showed cytotoxicities against melanoma cell line A375 and gastric cancer cell line MGC-803 with IC_50_ values of 22.76 and 36.65 μM, respectively, superior to those of referenced adriamycin [37]. 4-deoxy-*ε*-pyrromycinone, found in endophytic *Streptomyces* sp. Lz531 isolated from branch tissues of *Maytenus hookeri* well-known for producing anticancer compounds [38], was obtained again in the extract of LRE541. This study firstly demonstrated anticancer activities of 4-deoxy-*ε*-pyrromycinone against A549, HepG2, SW1990, and RKO. Epsilon-pyrromycinone, an anthracycline antibiotic, the yield of which was once increased 12-folds by strain improvement of *Streptomyces galilaeus* [40]. The antineoplastic activities of epsilon-pyrromycinone towards cancer cell lines were also evaluated for the first time in the study.

### Evaluation of preliminary anticancer mechanism of the LRE541 extract

It is well known that apoptosis and necrosis are two patterns of cell death [41, 42]. Compared to necrosis, an abnormal form of cell death, cell apoptosis regulated by various intra- and extracellular signals and governed by several genes, plays an important role in stress responses, control of normal cell proliferation and development of an organism [43, 44]. Tumorigenesis is closely related to anti-apoptotic pathways [4], and drug induced apoptosis of malignant cells is an efficient strategy in cancer therapy [4, 45]. Our data presented that the LRE541 extract from the endophytic *Streptomyces* sp. LRE541 validly inhibited the cell viabilities of RKO and 7901 predominantly through the induction of apoptosis in a dose-dependent manner. Apparently, the apoptosis patterns between the two cell lines were remarkably diverse, which suggested distinct mechanisms of the secondary metabolites actions occurring in the two cancer cell lines. Furthermore, previous studies have shown that the cell cycle is likewise intimately associated with the tumorigenesis. Pathological or physiological apoptotic stimuli would greatly affect cell cycle progression, and disorder of cell cycle regulators is a common property of human cancer, which signifies that regulation of cell cycle progression in cancer cells is taken for an available method in the treatment of human malignancies [46, 47]. In this study, the LRE541 extract dramatically inhibited the cell proliferation of RKO and 7901 in a dose-dependent manner by inducing S phase arrest of cell cycle and apoptosis in vitro. Collectively, chemotherapeutics with greater therapeutic efficiency and fewer side effects are of utmost desirability, and drug induced cancer cell death mode plays a vital role in chemotherapy.

## Conclusion

This study characterized the endophytic *Streptomyces* sp. LRE541 isolated from the root tissues of *L. davidii* var. *unicolor* (Hoog) Cotton and examined the cytotoxic activities of secondary metabolites of the isolate against a panel of human malignant cell lines, further detecting the cell apoptosis and cell cycle arrest of RKO and 7901 by flow cytometry revealed a primary mechanism underlying the biological action of the secondary metabolites and might shed light on the potential application of the metabolites in the therapy of RKO and 7901 cell lines. The chemical profile of the LRE541 extract detected by the UHPLC-MS/MS analysis revealed the presence of antitumor- and antimicrobial-compounds in the extract. Further chemical investigation of the extract of *Streptomyces* sp. LRE541 led to discovering one prenylated indole diketopiperazine (DKP) alkaloid, elucidated as neoechinulin A, a known antitumor agent; two anthraquinones, 4-deoxy-*ε*-pyrromycinone and epsilon-pyrromycinone both displaying anticancer activities.

## Materials and methods

### Sample collection and actinomycetes isolation

During March 2017, thirty healthy roots of 3-year-old *L. davidii* var. *unicolor* (Hoog) Cotton were randomly selected from the lily planting farm of Shaojia Shan (35° 57′ 50.73″ N, 103° 48′ 39.69″ E, H: 1868 m) in Qilihe District, Lanzhou City, Gansu Province, China. The plant roots were dug out carefully to ensure its integrity, then kept in aseptic plastic bags at 4 °C and processed within 24 h after collection. After being washed in running water, the surfaces of the roots were sterilized by sequential immersion in 0.1% (v/v) Tween 20 for 5 min, 75% (v/v) alcohol for 5 min, a solution of 2% (v/v) sodium hypochlorite for 5 min, and 10% (w/v) sodium bicarbonate solution for 5 min. Samples were washed in sterile distilled water at least three times to remove surface sterilization agents. Meanwhile, an aliquot (0.2 mL) of the last washing water was spread on agar plates and incubated at 28 °C for 7 days to confirm surface sterilization. The surface-sterilized roots were then aseptically sectioned by a commercial blender and spread onto the Gauze’s No. 1 media (20 g of soluble starch, 1 g of KNO_3_, 0.5 g of K_2_HPO_4_, 0.5 g of MgSO_4_·7H_2_O, 0.5 g of NaCl, 0.01 g of FeSO_4_·7H_2_O, 20 g of agar, pH 7.2–7.4; 121 °C, 20 min) supplemented with cycloheximide (25 mg/mL) and nystatin (10 mg/mL), followed by incubation at 23 °C for up to 2 weeks.

The morphology and growth of suspected actinomycetes were observed every day. Various colony characteristics such as powdery or leathery appearance with concave, convex, crumpled or flate surface, and pigment production were recorded. Representative isolates of 15 colonies with visually distinctive morphologies were selected from 50 initially recovered colonies and re-purified for further studies.

### Preliminary screening of endophytic isolates for antimicrobial activities

All 15 pure isolates were screened for antimicrobial activities by the double layer agar method [29]. Spore suspension of each isolate was inoculated on the Gauze’s No. 1 medium and incubated at 23 ℃ for 7 days, then overlaid with 5 mL of 0.6% (w/v) soft nutrient agar seeded with 500 μL of the culture of indicator microorganisms with a turbidity of 0.5 McFarland (10^7^–10^8^ CFU/mL), including Methicillin-resistant *Staphylococcus aureus* (MRSA) ATCC25923, *Escherichia coli* ATCC25922, *Pseudomonas aeruginosa* ATCC27853, *Candida albicans* ATCC66415, *Staphylococcus saprophyticus* (clinical isolate), *Enterococcus faecalis* (clinical isolate), *Diplococcus pneumoniae* (clinical isolate). Then, the overlaid plates were incubated at 28 °C for 24 h, the apparent inhibition zone around each isolate was recorded as positive for antimicrobial activity [29]. Plates with the same medium without actinomycete-like isolates but simultaneously inoculated with the indicator microorganisms were maintained as controls.

### Antimicrobial assay of isolate LRE541

The actinomycete-like isolate that exhibited an apparent inhibition zone against all the tested pathogenic microorganisms was furtherly evaluated by disc diffusion assay [48]. Briefly, isolate LRE541 was inoculated into the Gauze’s No. 1 liquid medium performing a small-scale fermentation for 7 days. The sterile filter paper discs (6 mm diameter) were impregnated into the culture filtrate of LRE541 overnight and air-dried, then placed onto the plates loaded with indicator microorganisms, incubated at 28 °C for 24 h. The discs loaded with the sterile Gauze’s No. 1 liquid filter without inoculating LRE541 were used for controls. The mean value of diameters for the zone of inhibition was calculated from the triplicate assays.

### Morphological and physiological characteristics of LRE541 isolate

To investigate the morphological and cultural characteristics of isolate LRE541, pure culture of LRE541 was examined every day grown on various international *Streptomyces* project (ISP) media. Micromorphology and sporulation of the culture was examined by the light microscopy (Olympus IX71) using the inclined coverslip technique [49] on the Gauze’s No. 1 medium for 7 days. The aerial mycelia and spores were observed under the scanning electron microscopy (SEM) (Hitachi S-3400N) after 14 days of growth on the Gauze’s No. 1 medium. Physiological characteristics such as extracellular enzyme activity, carbon/nitrogen source utilization, and temperature/pH tolerance were evaluated following the methods depicted in the Bergey’s Manual of Systematic Bacteriology [50] and the ISP [51].

### 16S rRNA gene sequencing and phylogenetic analysis

The genomic DNA (gDNA) of isolate LRE541 was extracted as described by Orsini et al. [52] with minor adjustment. The universal bacterial primers targeted 16S rDNA, 27 F (5′-AGAGTTTGATCCTGGCTCAG-3′) and 1525 R (5′-AAGGAGGTGATCCAGCCGCA-3′), were used for polymerase chain reaction (PCR) amplification following the manufacture’s protocol (Takara, Japan) with optimized adjustment. The checked PCR products were directly subjected to cycle sequencing using an ABI3100 automated sequencer (Beijing Sangon Biotech, Beijing, China). The sequenced 16S rRNA gene of isolate LRE541 was matched with the nearest gene sequences of *Streptomyces* spp. retrieved from a public database using the EzBioCloud tool with Clustal W program. The phylogenetic tree was constructed by using the Maximum Likelihood method [29] and *p*-distance model with bootstrap analysis of 1000 replicates [53] in the MEGA X package.

The 16S rRNA gene sequence of isolate LRE541 had been submitted to the GenBank nucleotide sequence databases under accession no. MK138546.

### Fermentation and extraction of secondary metabolites from LRE541

Isolate LRE541 cultured on a slant agar medium was inoculated into a 500 mL Erlenmeyer flask containing 100 mL of the seed medium consisting of 15 g/L soluble starch, 10 g/L soybean powder, 1 g/L NaCl, 5 g/L glucose, 5 g/L tryptone, and 5 g/L CaCO_3_ (pH 7.3). The seed media were cultivated on a rotary shaker (150 rpm) at 28 °C for 3 days. Then 11 mL of seed broth was transferred into a 1000-mL Erlenmeyer flask containing 500 mL Gause’s liquid medium, and incubated at 28 °C, 150 rpm for 9 days. After the fermentation process, the biomass was discarded by centrifugation at 10,000×*g* for 20 min while the supernatant was harvested and extracted three times with an equal volume of ethyl acetate. Then the ethyl acetate fractions were concentrated at 40 °C in a rotary vacuum distillation apparatus and dissolved in DMSO (1 mg/mL) for the investigation of antitumor activities.

### In vitro cytotoxic assay of the extract from LRE541

#### Cell culture

The antitumor activity of the LRE541 extract was examined against a wide variety of cell lines, including nine human cancer cell lines (HepG-2, SW-1190, CAL-27, 7901, RKO, MCF-7, Hela, K562, A549) and one normal human pulmonary artery endothelial cell line (HPAEC), which were purchased from the Shanghai Institute of Biochemistry and Cell Biology, Chinese Academy of Sciences. The cells were incubated in RPMI-1640 medium supplemented with 10% (v/v) fetal bovine serum (FBS), 2 mM glutamine and 100 units/mL streptomycin–penicillin, then maintained in a humidified atmosphere of 5% CO_2_ at 37 °C.

#### Cytotoxicity assay

The cell survival rate was evaluated using the MTT assay [31]. In short, the cells were seeded at a density of 1 × 10^4^ cells/well in 96-well plates for 24 h, then the medium was replaced with a fresh medium containing different concentrations of the LRE541 extract for 48 h. Cells treated with DMSO alone were set as negative controls, and cis-platinum was used as the positive control. Later, 10 μL MTT (5 mg/mL) reagent was added to each well and incubated for an additional 4 h at 37 °C. Absorbance (490 nm) of the medium was measured using a microplate reader (Thermo Scientific Multiskan GO, Finland).

#### Cell apoptosis analysis

RKO and 7901 cells were seeded in 6-well plates for 24 h, then incubated with LRE541 extract of various concentrations (0, 0.5, 1, and 2 μg/mL) for 48 h. Then, both of the cells were collected and washed with phosphate-buffered saline (PBS) (0.01 M; pH 7.4) three times. Afterwards, cell samples were stained with fluorescein 5-isothiocyanate (FITC)-conjugated annexin V and propidium iodide (PI) following the manufacturer’s instructions (Zoman Biotech, Beijing, China). Data was obtained and analyzed using a FACS-Canto flow cytometer (BD Biosciences, San Jose, CA, USA) with FlowJo software.

#### Cell cycle analysis

Cell cycle analysis was also performed by flow cytometry [54]. In brief, RKO and 7901 cells were plated in 6-well plates for 24 h and then incubated with the LRE541 extract of various concentrations (0, 0.5, 1, and 2 μg/mL) for 48 h. Then, the cells were harvested and washed with PBS three times, and the percentages of cells in the G_0_/G_1_, S, and G_2_/M phases were analyzed using the FACS-Canto flow cytometer (BD Biosciences, San Jose, CA, USA) in the presence of propidium iodide buffer (50 μg/mL; pH 7.4) with RNase (100 μg/mL; pH 7.4) (Zoman Biotech, Beijing, China).

### Metabolite profiles by the UHPLC-MS/MS analysis

The LRE541 extract was subjected to a Vanquish UHPLC system equipped with an Orbitrap Q Exactive series mass spectrometer (Thermo Fisher) for the metabolite profile analysis. The processed samples were injected onto a Hyperil Gold column (100 × 2.1 mm, 1.9 μm) using a 16-min linear gradient at a flow rate of 0.2 mL/min. The eluents for the positive polarity mode were eluent A (0.1% formic acid in water) and eluent B (methanol), and for the negative polarity mode were eluent A (5 mM ammonium acetate, pH 9.0) and eluent B (methanol). The solvent gradient was set as follows: 2% B, 1.5 min; 2–100% B, 12.0 min; 100% B, 14.0 min; 100–2% B, 14.1 min; 2% B, 17 min. Q Exactive series mass spectrometer was operated in the positive/negative polarity mode with a spray voltage of 3.2 kV, capillary temperature of 320 °C, sheath gas flow rate of 35 arb, and aux gas flow rate of 10 arb.

### Purification and characterization of bioactive metabolites from LRE541 extract

The ethyl acetate extract of LRE541 was separated and purified on an HP-20 macroporous resin (Mitsubishi, Japan) column and eluted with gradient mixtures of H_2_O–EtOH (70:30, 50:50, 20:80) to give three fractions (A–C). After evaporation of the menstruum in vacuo, the fraction C (0.5 g) was resolved by chromatography on a silica gel column eluted with CHCl_3_/EtOAc mixtures with a growing polarity (25:1–1:1, v/v) to obtain six fractions (C1–6). Bioactivity assays (in vitro antitumor activity) indicated that C3 (CHCl_3_/EtOAc, 10:1) and C4 (CHCl_3_/EtOAc, 7:1) fractions were cytotoxic in vitro. The active fractions (C3 and 4) were repeatedly purified and separated on Sephadex LH-20 (CHCl_3_:MeOH, 1:1) by semi-preparative HPLC (NP7001C, C18, 5 μm, 250 × 10 mm inner diameter; Hanbon Sci. & Tech.) to afford compounds (**1**)–(**3**) (1, 1.2, 1.5 mg of each).

Structural identification of the purified metabolites was elucidated on Bruker DRX-400 spectrometer (Bruker, Rheinstetten, Germany) by using spectroscopic techniques for ^1^H and ^13^C (400 MHz for ^1^H and 100 MHz for ^13^C). Chemical shifts were reported in ppm. (*δ*), using residual CHCl_3_ (*δ*_H_ 7.26 ppm; *δ*_C_ 77.0) and (CH_3_)_2_ CO (*δ*_H_ 2.05 ppm; *δ*_C_ 29.84) as an internal standard, with coupling constants (*J*) in Hz. Moreover, the HMBC and HSQC techniques were also performed for supporting the ^1^H and ^13^C spectroscopic analysis.

### Statistical analysis

Data were expressed as the means ± SD for at least three independent experiments. SPSS software was applied to perform the statistical analysis, and the statistical differences between the two groups were assessed by Student’s t-test. *P* < 0.05 was used as the criterion for statistical significance.

## Supplementary Information

**Additional file 1: Table S1.** Antimicrobial activities of the actinomycete-like isolates from the root tissues of *Lilium davidii* var*. unicolor* (Hoog) Cotton. **Table S2.** Chemical constituents of antitumor-(1–39), antioxidant-(40–49), and antimicrobial-(50–65) compounds identified in the LRE541 extract by the UHPLC-MS/MS analysis. **Figure S1.** Total iron chromatography of the LRE541 extract by the UHPLC-MS/MS analysis. **Figure S2.** Chemical structures of the antitumor (1–39)-, antioxidant (40–49)-, and antimicrobial (50–65)-compounds from the LRE541 extract. **Figure S3.** ^13^C NMR spectrum of epsilon-pyrromycinone in CDCl_3_ (100 MHz). **Figure S4.** ^1^H NMR spectrum of Epsilon-pyrromycinone in CDCl_3_ (400 MHz). **Figure S5.** ^13^C NMR spectrum of 4-deoxy-ε-pyrromycinone in CDCl_3_ (100 MHz). **Figure S6.** ^1^H NMR spectrum of 4-deoxy-ε-pyrromycinone in CDCl_3_ (400 MHz). **Figure S7.** HMBC spectrum of 4-deoxy-ε-pyrromycinone in CDCl_3_. **Figure S8.** HSQC spectrum of 4-deoxy-ε-pyrromycinone in CDCl_3_. **Figure S9.** ^13^C NMR spectrum of Neoechinulin A in CDCl_3_ (100 MHz). **Figure S10.** ^1^H NMR spectrum of Neoechinulin A in CDCl_3_ (400 MHz).

## Data Availability

All data generated or analyzed during the study were included in this paper and its Additional file 1.

## Abbreviations

PBS: Phosphate buffered saline
MTT: 3-(4,5-Dimethylthiazol-2-yl)-2,5-diphenyl tetrazolium bromide
DMSO: Dimethyl sulfoxide
SEM: Scanning electron microscopy
ANOVA: Analysis of variance
PI: Propidium iodide
FBS: Fetal bovine serum
IC_50_: Inhibition concentration of 50% growth
EtOAc: Ethyl acetate
rpm: Rotations per minute
TLC: Thin-layer chromatography
HPLC: High performance liquid chromatography
w/v: Weight/volume
v/v: Volume/volume
