# Supplementary material for: Evaluation of the anticarcinogenic potential of the endophyte, Streptomyces sp. LRE541 isolated from Lilium davidii var. unicolor (Hoog) Cotton
Source: Microb Cell Fact. 2021 Dec 4;20:217. doi: 10.1186/s12934-021-01706-z (PMC8643024; doi:10.1186/s12934-021-01706-z)
Supplement: Supplementary file 1 — Additional file 1: Table S1. Antimicrobial activities of the actinomycete-like isolates from the root tissues of Lilium davidii var. unicolor (Hoog) Cotton. Table S2. Chemical constituents of antitumor-(1–39), antioxidant-(40–49), and antimicrobial-(50–65) compounds identified in the LRE541 extract by the UHPLC-MS/MS analysis. Figure S1. Total iron chromatography of the LRE541 extract by the UHPLC-MS/MS analysis. Figure S2. Chemical structures of the antitumor (1–39)-, antioxidant (40–49)-, and antimicrobial (50–65)-compounds from the LRE541 extract. Figure S3. 13C NMR spectrum of epsilon-pyrromycinone in CDCl3 (100 MHz). Figure S4. 1H NMR spectrum of Epsilon-pyrromycinone in CDCl3 (400 MHz). Figure S5. 13C NMR spectrum of 4-deoxy-ε-pyrromycinone in CDCl3 (100 MHz). Figure S6. 1H NMR spectrum of 4-deoxy-ε-pyrromycinone in CDCl3 (400 MHz). Figure S7. HMBC spectrum of 4-deoxy-ε-pyrromycinone in CDCl3. Figure S8. HSQC spectrum of 4-deoxy-ε-pyrromycinone in CDCl3. Figure S9. 13C NMR spectrum of Neoechinulin A in CDCl3 (100 MHz). Figure S10. 1H NMR spectrum of Neoechinulin A in CDCl3 (400 MHz). [file 12934_2021_1706_MOESM1_ESM.docx]

# Evaluation of the anticarcinogenic potential of the endophyte, *Streptomyces* sp. LRE541 isolated from *Lilium davidii* var*.* *unicolor* (Hoog) Cotton

Aiai Ma, Kan Jiang, Bin Chen, Shasha Chen, Xinge Qi, Huining Lu, Junlin Liu, Xuan Zhou, Tan Gao, Jinhui Li ＆ Changming Zhao^*^

*Corresponding author: Tel: 13893496972.

*E-mail address*: [zhaochm@lzu.edu.cn](mailto:zhaochm@lzu.edu.cn)

Table S1 Antimicrobial activities of the actinomycete-like isolates from the root tissues of *Lilium davidii* var*.* *unicolor* (Hoog) Cotton

| Isolate no. | Antimicrobial activity | | | | | | |
| --- | --- | --- | --- | --- | --- | --- | --- |
|  | *S. aureus* | *D. pneumoniae* | *E. faecalis* | *S. saprophyticus* | *E. coli* | *P. aeruginosa* | *C. albicans* |
| LRE911 | － | － | － | － | － | － | － |
| LRE141 | － | － | － | － | － | － | － |
| LRE545 | + | + | － | + | + | － | － |
| LRE843 | － | － | － | － | － | － | － |
| LRE842 | － | － | － | － | － | － | － |
| LRE841 | － | － | － | － | － | － | － |
| LRE844 | － | － | － | － | － | － | － |
| LRE948 | － | － | － | － | － | － | － |
| LRE344 | － | － | － | － | － | － | － |
| LRE443 | － | － | － | － | － | － | － |
| LRE546 | － | － | － | － | － | － | － |
| LRE743 | － | － | － | － | － | － | － |
| LRE744 | － | － | － | － | － | － | － |
| LRE442 | － | － | － | － | － | － | － |
| LRE142 | － | － | － | － | － | － | － |
| LRE811 | － | － | － | － | － | + | － |
| LRE143 | － | － | － | － | － | － | － |
| LRE543 | － | － | － | － | － | － | － |
| **LRE541** | **+** | **+** | **+** | **+** | **+** | **+** | **+** |
| LRE2234 | － | － | － | － | － | － | － |

+: antimicrobial activity; －: no antimicrobial activity

Table S2 Chemical constituents of antitumor- (1-39), antioxidant- (40-49), and antimicrobial- (50-65) compounds identified in the LRE541 extract by the UHPLC-MS/MS analysis

| Mode | No. | Constituents | Molecular formula | *m/z* | Retention time (min) | Relative ratio (%) |
| --- | --- | --- | --- | --- | --- | --- |
| ESI (−) | 1 | Ferulic acid | C_10_H_10_O_4_ | 194.05786 | 8.241 | 15.3965 |
|  | 2 | 4-Hydroxybenzylalcohol | C_7_H_8_O_2_ | 124.05242 | 8.554 | 0.6811 |
|  | 3 | Formononetin | C_16_H_12_O_4_ | 268.07325 | 13.577 | 0.5133 |
|  | 4 | Sinapic acid | C_11_H_12_O_5_ | 224.06869 | 8.077 | 0.2469 |
|  | 5 | (+/-)-Equol | C_15_ H_14_O_3_ | 242.09479 | 9.867 | 0.1788 |
|  | 6 | 4-Hydroxycoumarin | C_9_H_6_O_3_ | 162.03198 | 6.413 | 0.0484 |
|  | 7 | Resveratrol | C_14_H_12_O_3_ | 228.07894 | 9.699 | 0.015 |
|  | 8 | Catechin | C_15_H_14_O_6_ | 290.07954 | 10.018 | 0.009 |
|  | 9 | Naringenin | C_15_H_12_O_5_ | 272.06889 | 9.903 | 0.003 |
|  | 10 | Ursolic acid | C_30_H_48_O_3_ | 456.36082 | 14.59 | 0.0002 |
|  | 40 | 4-Hydroxyphenylacetic acid^*^ | C_8_H_8_O_3_ | 152.047 | 8.944 | 5.77495 |
|  | 41 | Eugenol* | C_10_H_12_O_2_ | 164.084 | 11.048 | 1.73939 |
|  | 42 | Rosmarinic Acid^*^ | C_18_H_16_O_8_ | 360.085 | 9.712 | 0.0009 |
|  | 43 | Eriodictyol* | C_15_H_12_O_6_ | 288.064 | 7.485 | 0.0008 |
|  | 44 | 7-Methylguanosine* | C_11_H_17_N_5_O_5_ | 299.124 | 11.823 | 0.0003 |
|  | 50 | Lauric acid** | C_12_H_24_O_2_ | 200.178 | 13.361 | 1.1963 |
|  | 51 | trans-Cinnamic acid** | C_9_H_8_O_2_ | 148.05252 | 8.859 | 0.7326 |
|  | 52 | Coniferyl alcohol** | C_10_H_12_O_3_ | 180.07876 | 9.483 | 0.4579 |
|  | 53 | Citrinin** | C_13_H_14_O_5_ | 250.08446 | 8.533 | 0.1862 |
|  | 54 | Mycophenolic acid** | C_17_H_20_ O_6_ | 320.12639 | 9.934 | 0.0751 |
|  | 55 | Undecanoic acid** | C_11_H_22_ O_2_ | 186.16217 | 12.79 | 0.0358 |
|  | 56 | 2-Hydroxy-1,4-naphthoquinone** | C_10_H_6_O_3_ | 174.03177 | 6.412 | 0.0058 |
| ESI (+) | 11 | 4-Methylumbelliferone | C_10_H_8_O_3_ | 176.04741 | 8.625 | 2.2697 |
|  | 12 | 7-Hydroxycoumarine | C_9_H_6_O_3_ | 162.03168 | 7.999 | 0.4477 |
|  | 13 | Citral | C_10_H_16_O | 152.12002 | 10.117 | 0.2339 |
|  | 14 | 2-Oxindole | C_8_H_7_NO | 133.05268 | 8.707 | 0.2231 |
|  | 15 | Alternariol | C_14_H_10_O_5_ | 258.05156 | 10.075 | 0.2003 |
|  | 16 | 4-Phenylbutyric acid | C_10_H_12_O_2_ | 164.08382 | 11.336 | 0.1944 |
|  | 17 | Coumarin | C_9_H_6_O_2_ | 146.03678 | 8.096 | 0.1679 |
|  | 18 | Genistein | C_15_H_10_O_5_ | 270.05259 | 10.735 | 0.1268 |
|  | 19 | Cuminaldehyde | C_10_H_12_O | 148.08881 | 13.689 | 0.095 |
|  | 20 | Isorhapontigenin | C_15_H_14_O_4_ | 258.08888 | 9.087 | 0.0886 |
|  | 21 | 3-Acetoxyurs-12-en-23-oic acid | C_32_H_50_O_4_ | 476.38654 | 13.587 | 0.0639 |
|  | 22 | Shikonin | C_16_H_16_O_5_ | 288.09939 | 7.453 | 0.0321 |
|  | 23 | Glycocholic acid | C_26_H_43_NO_6_ | 465.30892 | 13.916 | 0.0291 |
|  | 24 | 18-β-Glycyrrhetinic acid | C_30_H_46_O_4_ | 470.33931 | 12.8 | 0.0236 |
|  | 25 | (+)-ar-Turmerone | C_15_H_20_O | 216.15145 | 10.875 | 0.0199 |
|  | 26 | Betulin | C_30_H_50_O_2_ | 442.38092 | 14.295 | 0.0172 |
|  | 27 | 5'-S-Methyl-5'-thioadenosine | C_11_H_15_N_5_O_3_S | 297.08953 | 6.514 | 0.0142 |
|  | 28 | D-(+)-Camphor | C_10_H_16_O | 152.12003 | 12.35 | 0.014 |
|  | 29 | D-Sphingosine | C_18_H_37_NO_2_ | 299.28214 | 12.019 | 0.0101 |
|  | 30 | Viramune | C_15_H_14_N_4_O | 266.11528 | 10.455 | 0.0083 |
|  | 31 | Diosmetin | C_16_H_12_O_6_ | 300.06322 | 10.742 | 0.0066 |
|  | 32 | 1-Caffeoylquinic Acid | C_16_H_18_O_9_ | 354.09499 | 7.112 | 0.0062 |
|  | 33 | Cryptotanshinone | C_19_H_20_O_3_ | 296.1418 | 11.047 | 0.0015 |
|  | 34 | β-Lapachone | C_15_H_14_O_3_ | 242.09442 | 11.613 | 0.0013 |
|  | 35 | Diosgenin | C_27_H_42_O_3_ | 396.30347 | 12.826 | 0.0011 |
|  | 36 | Nobiletin | C_21_H_22_O_8_ | 402.13108 | 12.035 | 0.0006 |
|  | 37 | Fisetin | C_15_H_10_O_6_ | 286.04747 | 10.069 | 0.0005 |
|  | 38 | Isoliquiritigenin | C_15_H_12_O_4_ | 256.0735 | 13.644 | 0.0004 |
|  | 39 | Veratramine | C_27_H_39_NO_2_ | 409.29756 | 11.481 | 0.0003 |
|  | 45 | Daidzein* | C_15_H_10_O_4_ | 254.058 | 10.19 | 3.09282 |
|  | 46 | Apocynin* | C_9_H_10_O_3_ | 166.063 | 8.871 | 2.2817 |
|  | 47 | Glycitein* | C_16_H_12_O_5_ | 284.068 | 10.314 | 0.4888 |
|  | 48 | (-)-Caryophyllene oxide* | C_15_H_24_O | 220.183 | 13.938 | 0.2325 |
|  | 49 | Cynaropicrin* | C_19_H_22_O_6_ | 346.141 | 9.674 | 0.0407 |
|  | 57 | Sorbic acid** | C_6_H_8_O_2_ | 112.05235 | 6.724 | 14.8589 |
|  | 58 | 3,5-Dimethoxybenzoic acid** | C_9_H_10_O_4_ | 182.058 | 6.835 | 2.7591 |
|  | 59 | Methyl cinnamate** | C_10_H_10_O_2_ | 162.06811 | 10.654 | 0.30 |
|  | 60 | trans-Cinnamaldehyde** | C_9_H_8_O | 132.05749 | 5.453 | 0.2613 |
|  | 61 | 2-Oxindole** | C_8_H_7_NO | 133.05268 | 8.707 | 0.2231 |
|  | 62 | 5-hydroxy-6,7-dimethoxy-2-phenyl-4H-chromen-4-one ** | C_17_H_14_O_5_ | 298.08368 | 10.553 | 0.0333 |
|  | 63 | 3,4-Dihydroxybenzaldehyde** | C_7_H_6_O_3_ | 138.02959 | 1.666 | 0.0189 |
|  | 64 | Cefradine** | C_16_H_19_N_3_O_4_S | 349.1028 | 13.521 | 0.0101 |
|  | 65 | Pleuromutilin** | C_22_H_34_O_5_ | 378.24019 | 11.912 | 0.0069 |

* denotes potential antitumor compounds; ** denotes antimicrobial compounds.


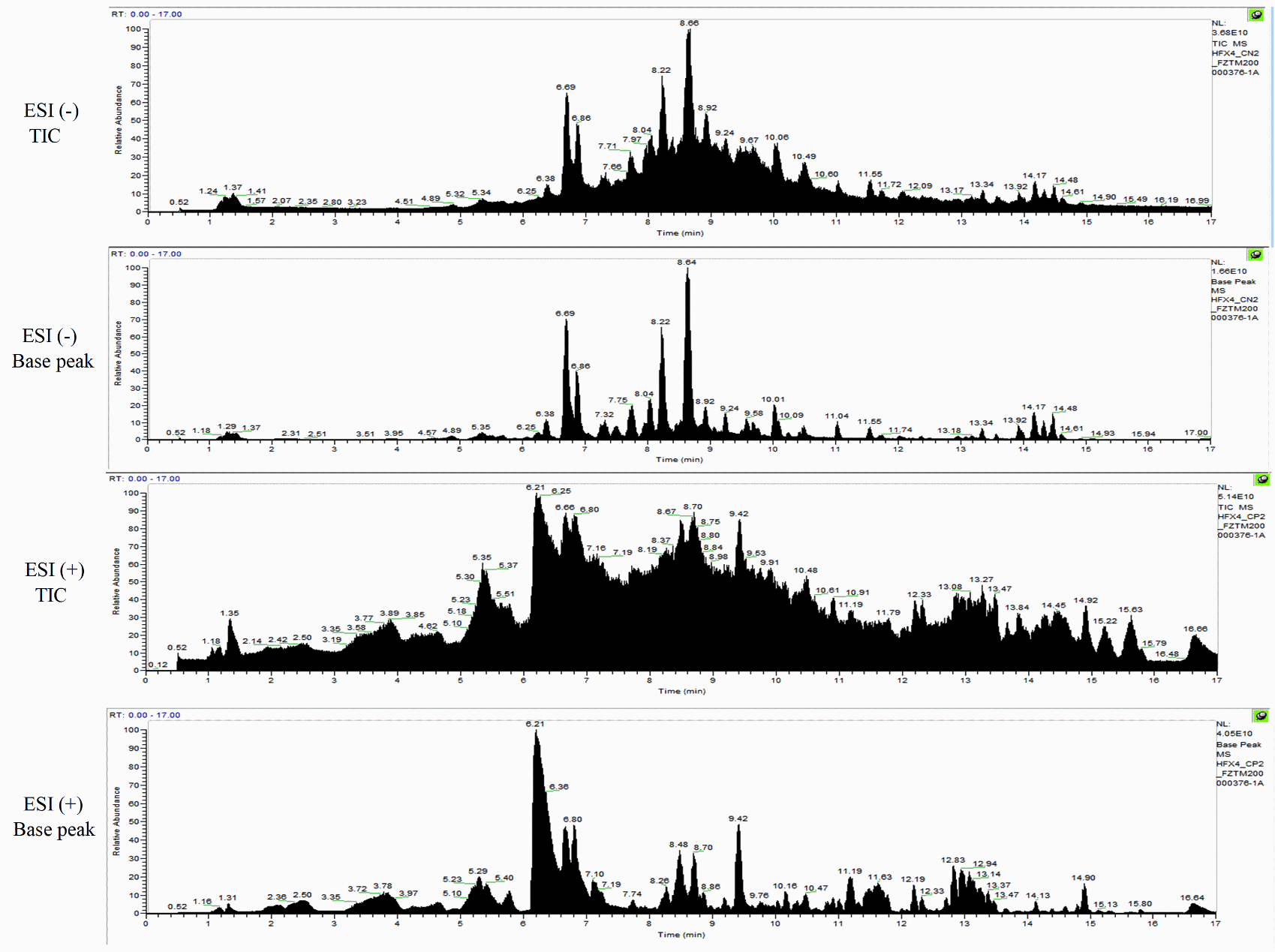


Fig. S1 Total iron chromatography of the LRE541 extract by the UHPLC-MS/MS analysis.


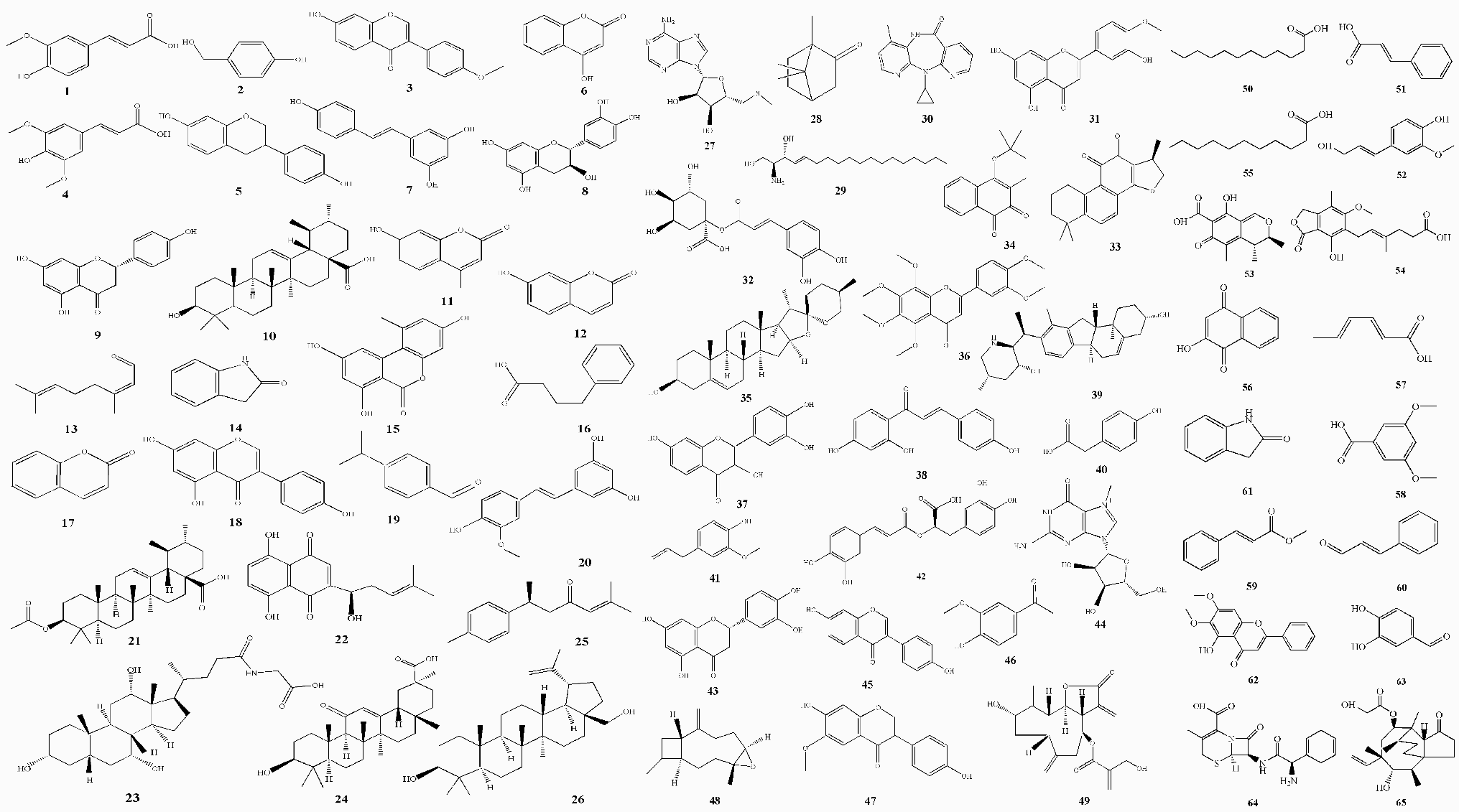


Fig. S2 Chemical structures of the antitumor (1-39)-, antioxidant (40-49)-, and antimicrobial (50-65)- compounds from the LRE541 extract.


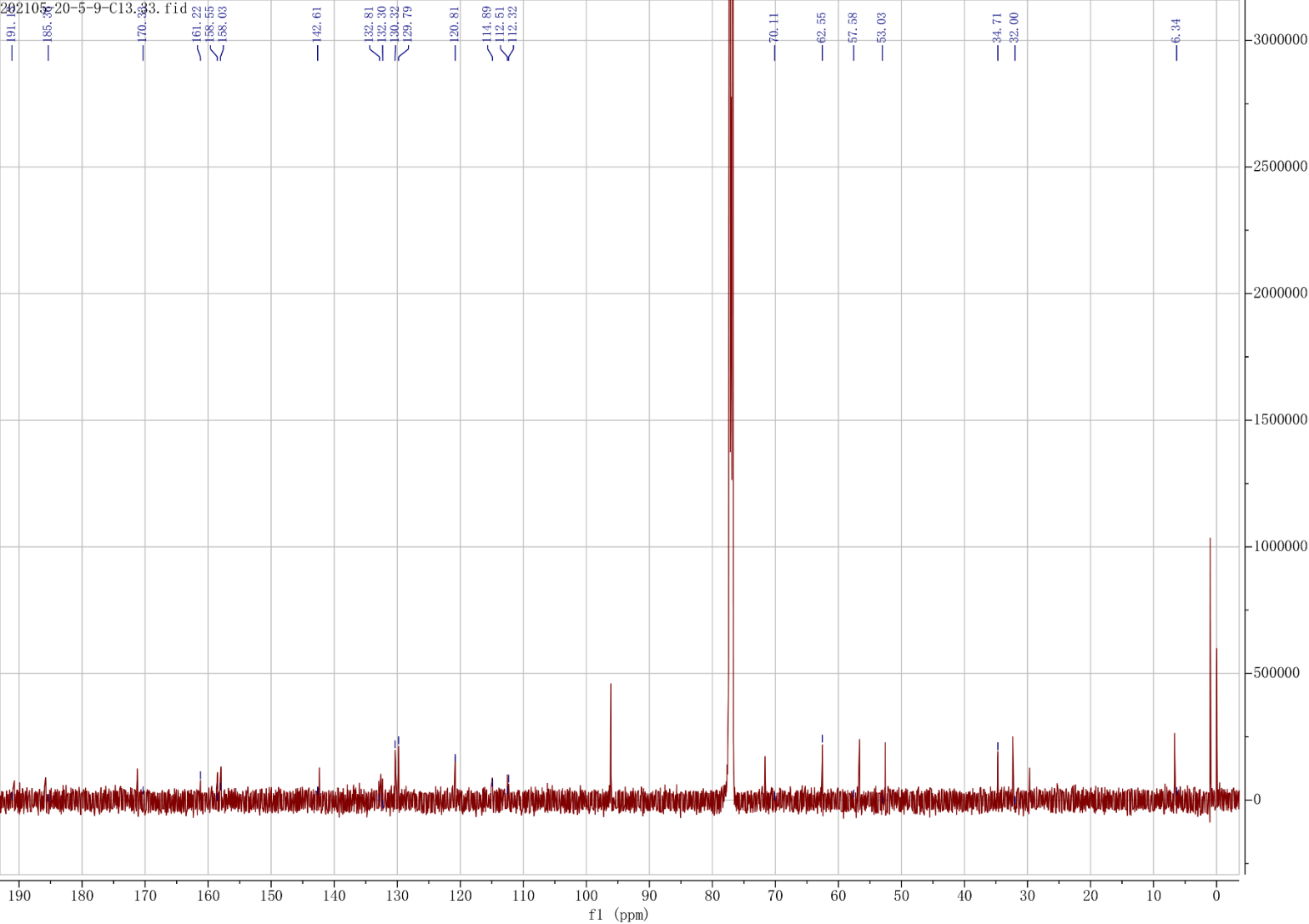


Fig. S3 ^13^C NMR spectrum of epsilon-pyrromycinone in CDCl_3_ (100 MHz).


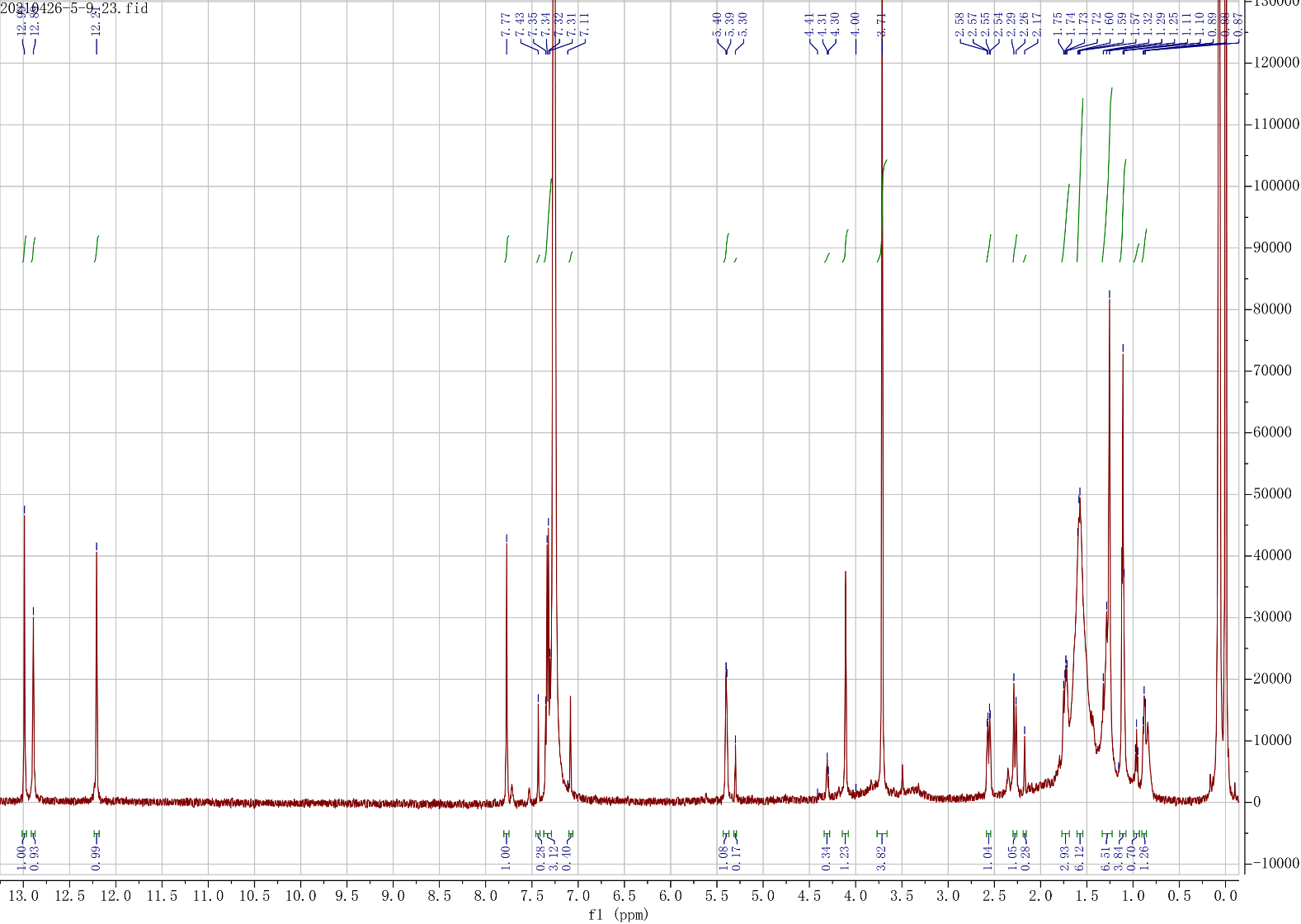


Fig. S4 ^1^H NMR spectrum of Epsilon-pyrromycinone in CDCl_3_ (400 MHz).


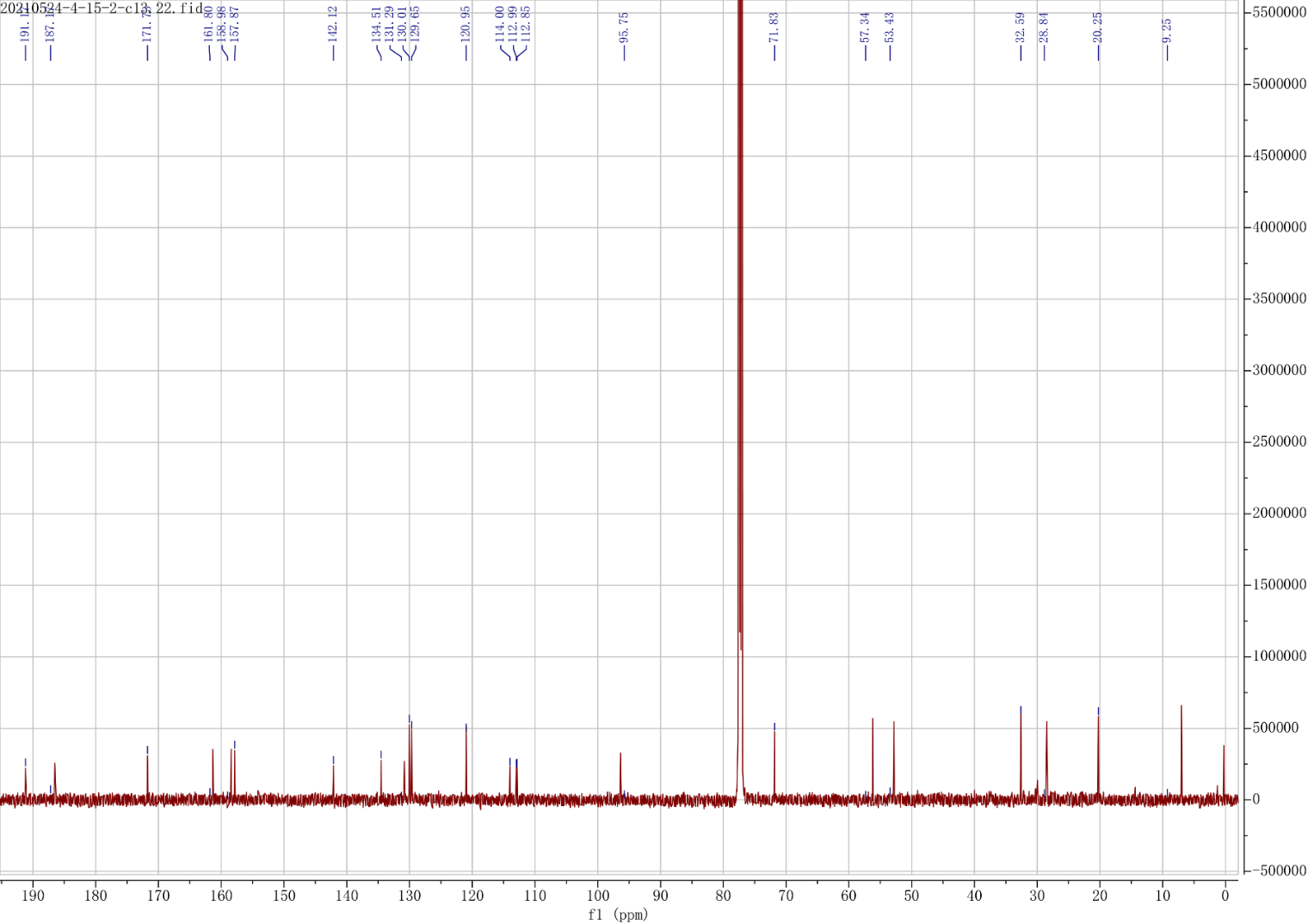


Fig. S5 ^13^C NMR spectrum of 4-deoxy-ε-pyrromycinone in CDCl_3_ (100 MHz).


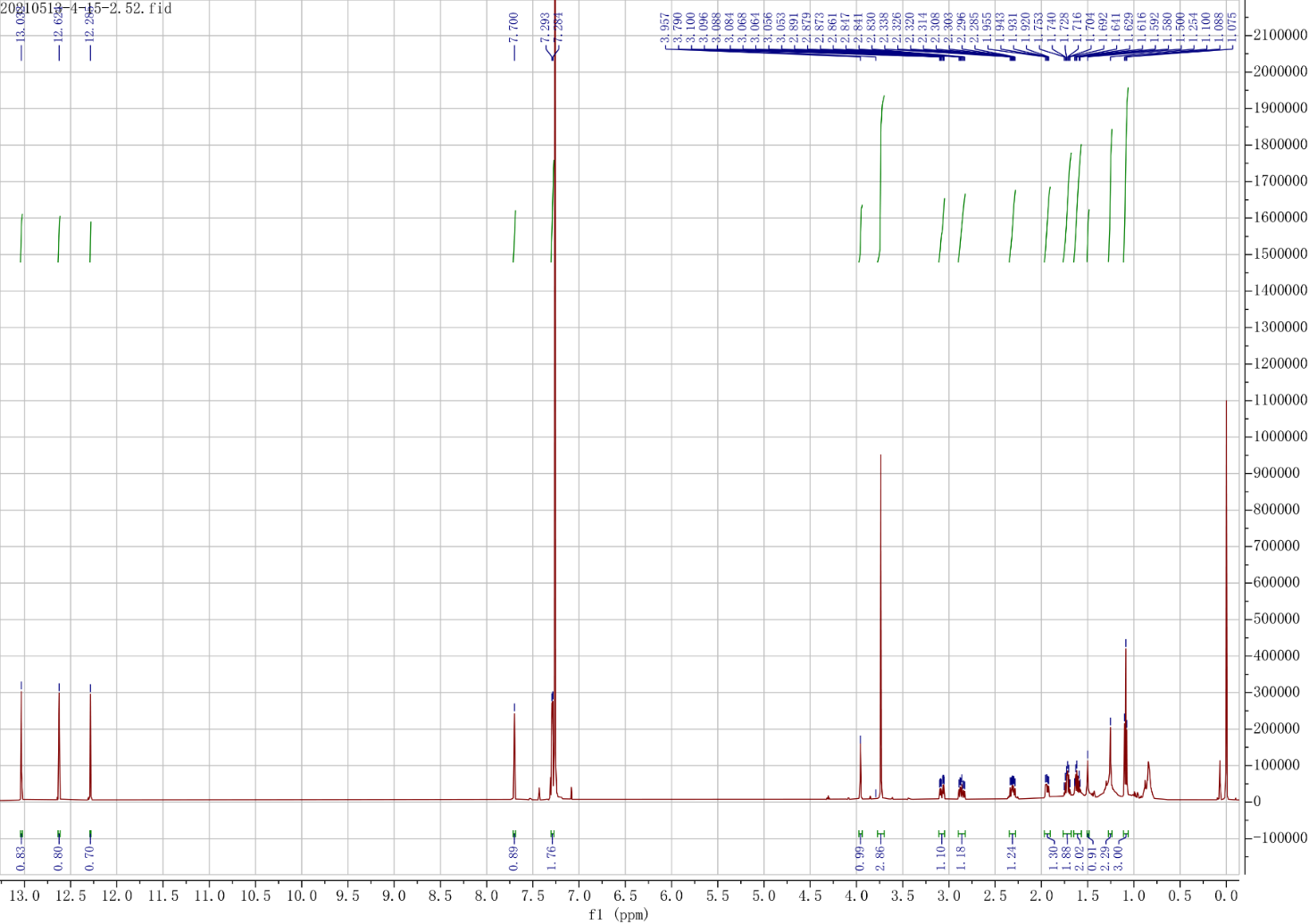


Fig. S6 ^1^H NMR spectrum of 4-deoxy-ε-pyrromycinone in CDCl_3_ (400 MHz).


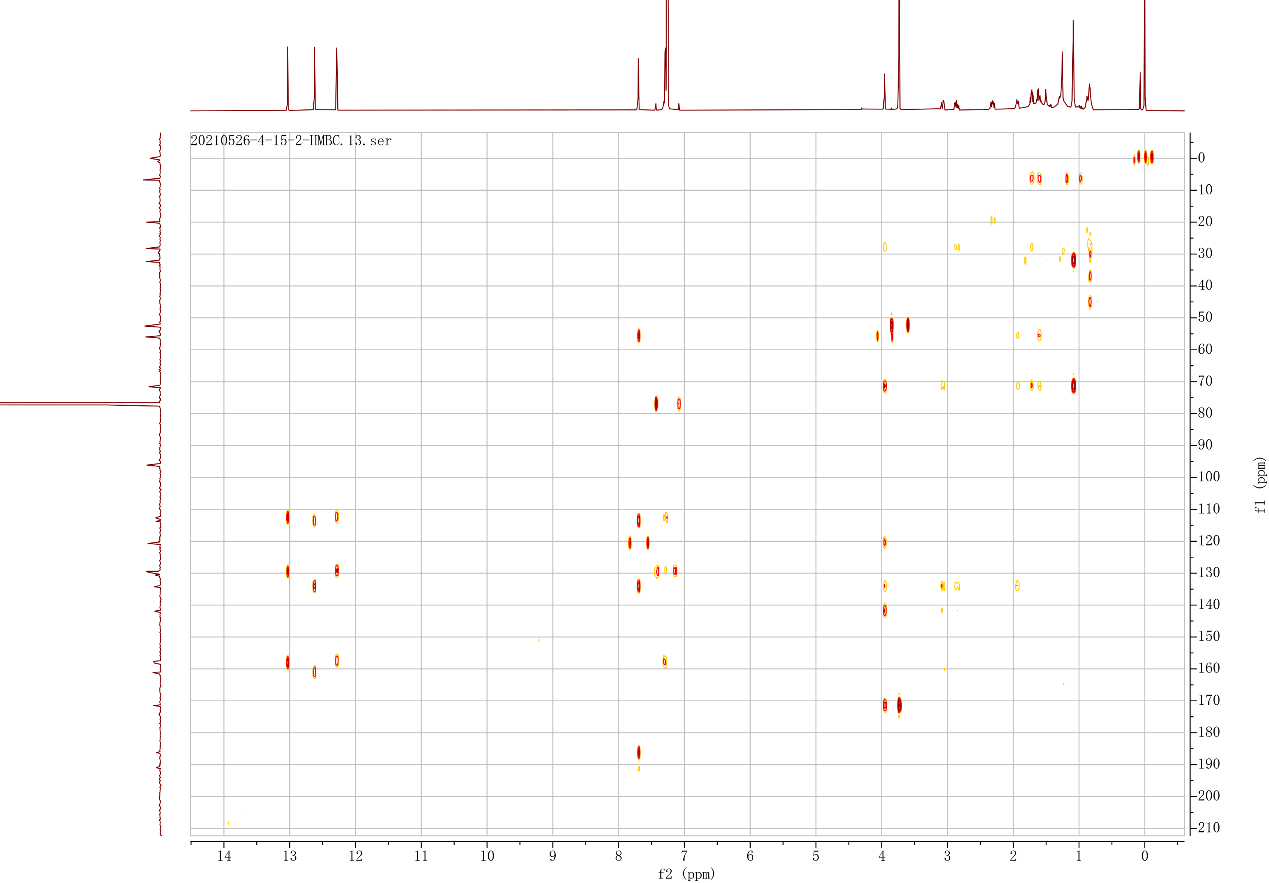


Fig. S7 HMBC spectrum of 4-deoxy-ε-pyrromycinone in CDCl_3._


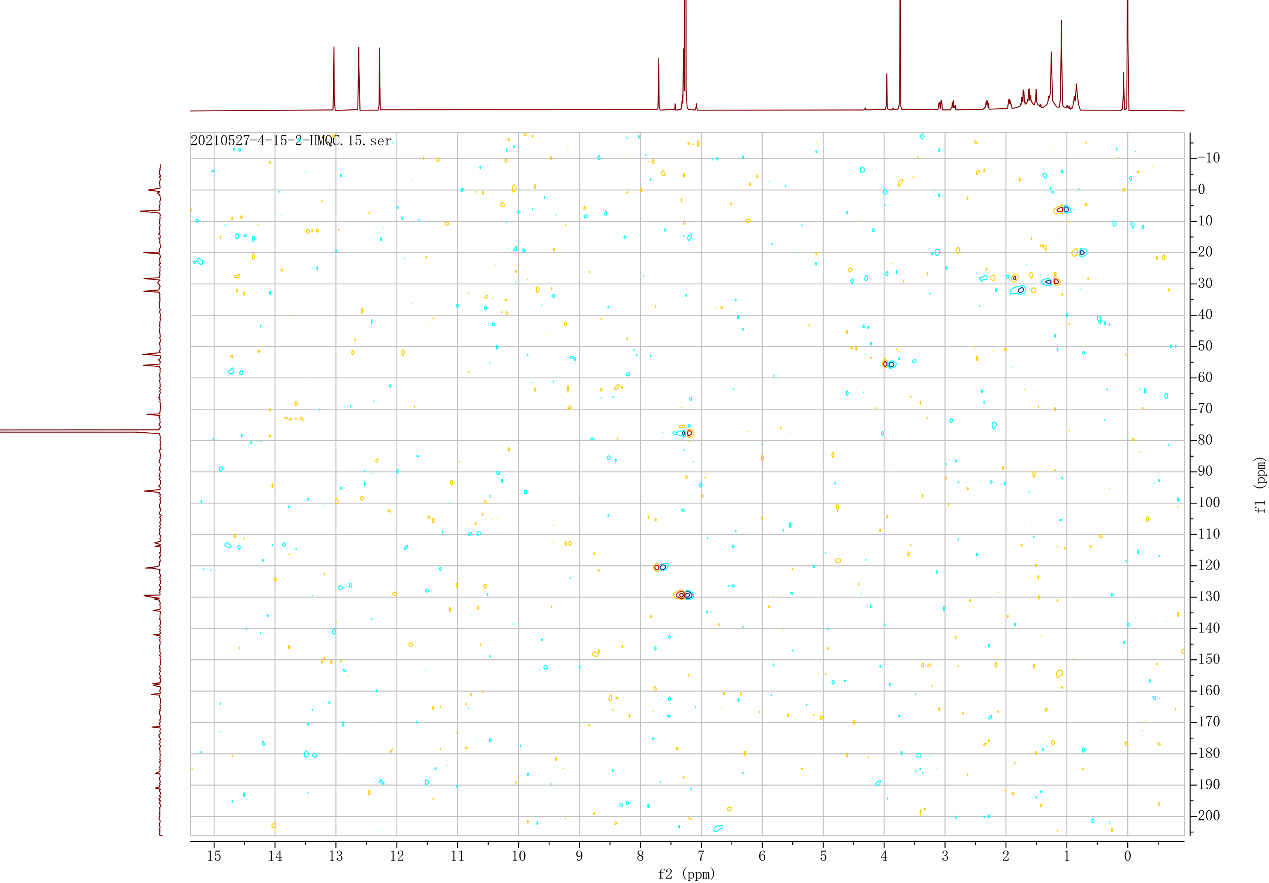


Fig. S8 HSQC spectrum of 4-deoxy-ε-pyrromycinone in CDCl_3._


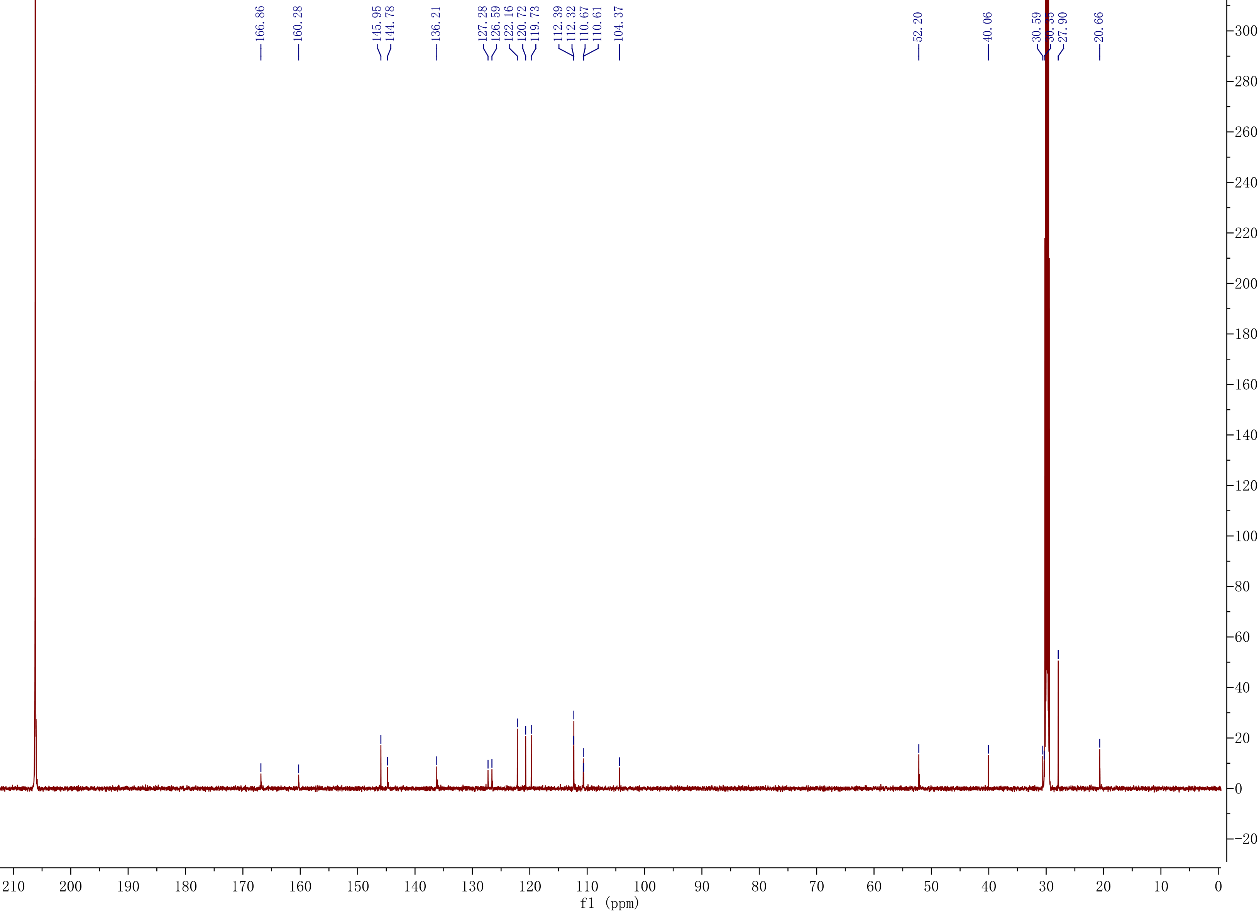


Fig. S9 ^13^C NMR spectrum of Neoechinulin A in CDCl_3_ (100 MHz).


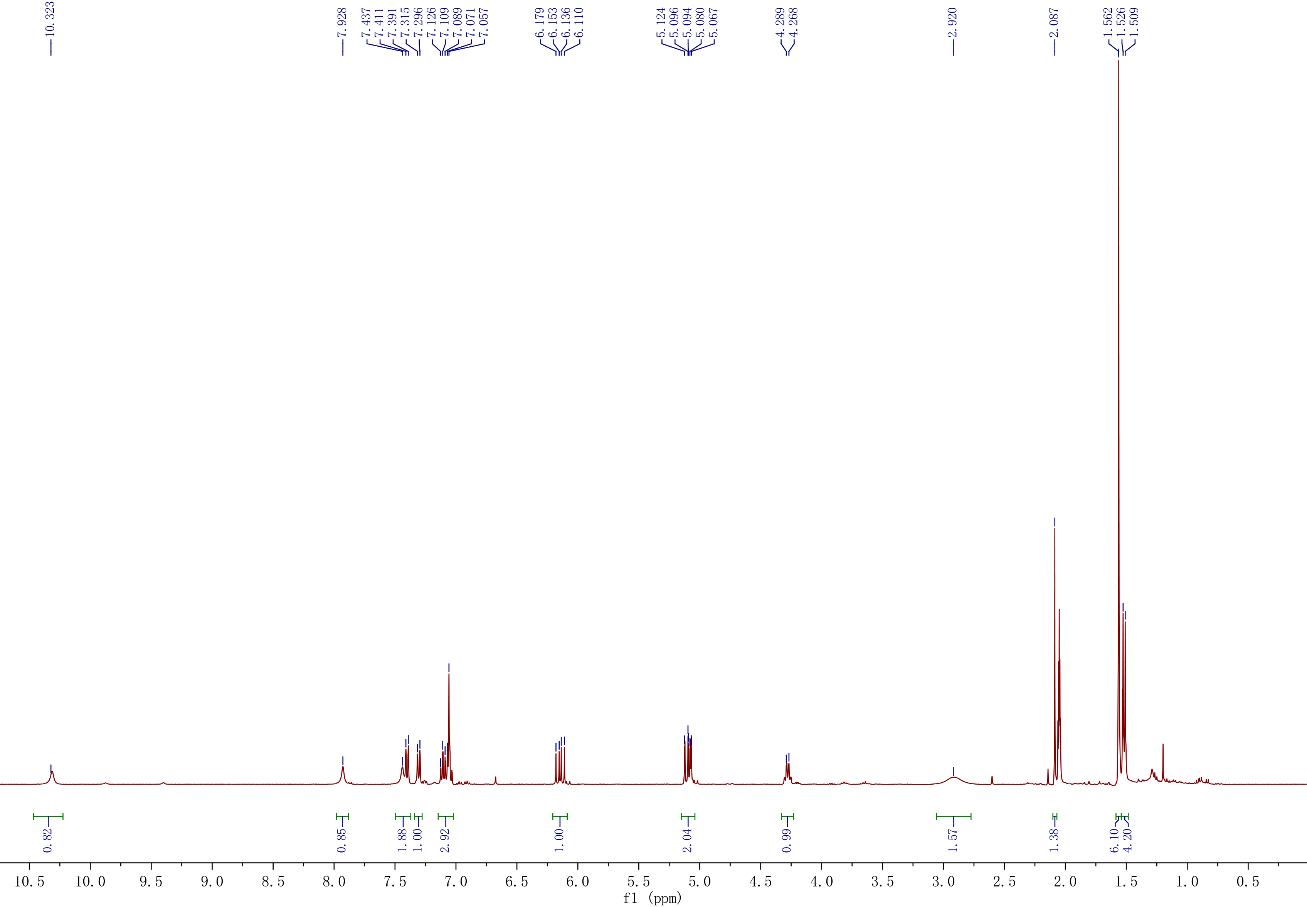


Fig. S10 ^1^H NMR spectrum of Neoechinulin A in CDCl_3_ (400 MHz).
